# Formulation of Recombinant Therapeutic Proteins: Technological Innovation, Regulations, and Evolution Towards Buffer-Free Formulations

**DOI:** 10.3390/pharmaceutics17091183

**Published:** 2025-09-11

**Authors:** Tomas Gabriel Bas

**Affiliations:** Escuela de Ciencias Empresariales, Universidad Católica del Norte, Coquimbo 1780000, Chile; tomas.bas@ucn.cl

**Keywords:** recombinant therapeutic proteins, buffer-free formulations, buffer formulations, protein formulation subcutaneous biologics, FDA/EMA regulatory frameworks, excipient safety, immunogenicity, intellectual property, IP, biosimilar formulation, pharmaceutical innovation

## Abstract

**Background/Objectives**: Formulating recombinant therapeutic proteins is essential to ensure their safety, efficacy, and stability. A growing trend in biopharmaceutical development is the move toward buffer-free formulations, which aim to reduce immunogenicity, improve tolerability, and simplify production. This review explores technological advances, regulatory perspectives, and safety considerations related to this shift. **Methods**: A systematic documentary review was conducted using the PSALSAR framework. Scientific publications, patents, and regulatory documents (2020–2025) were retrieved from PubMed, Scopus, Web of Science, and regulatory databases (FDA, EMA). Inclusion criteria focused on recombinant proteins, buffer-free formulations, and regulatory alignment. **Results**: The findings reveal an increasing adoption of self-buffering strategies in high-concentration subcutaneous biologics. Technologies such as Fc-fusion, PASylation, and XTENylation enhance stability without conventional buffers. Regulatory bodies are progressively accepting minimalist formulations, provided safety and biosimilarity are demonstrated. However, intellectual property barriers limit formulation transparency. A synthesis of recent FDA and EMA approvals illustrates this formulation evolution. **Conclusions**: Buffer-free formulations offer a promising alternative for therapeutic protein development by improving patient experience and reducing formulation complexity. They align with biosimilar goals and regulatory trends, although long-term transparency and safety assessments remain critical for widespread adoption.

## 1. Introduction

The field of recombinant therapeutic proteins has experienced a substantial evolution and growth since the advent of technological innovations based on recombinant DNA, which facilitates the combination of genetic material for the design of different varieties of proteins [1]. Recombinant DNA technology is based on the ability to manipulate DNA sequences to create new molecules composed of genetic material from two or more different organisms [2,3,4]. The formulation of recombinant therapeutic proteins represents a highly sophisticated and integral aspect of molecule development within the biopharmaceutical industry [5]. Recombinant therapeutic proteins are biologically modified substances derived from living cells to produce proteins with therapeutic effects [6]. These proteins are synthesized using recombinant DNA technology, allowing the insertion of specific genes into cells called hosts, usually bacteria or mammalian cells [7]. Upon introduction into the host, the vector provides the signals and mechanisms necessary for RNA transcription and its translation into protein [8,9]. This allows the mass production of specific and biologically active recombinant therapeutic proteins, such as hormones, cytokines, and monoclonal antibodies, which are used for the treatment of various chronic diseases such as diabetes, or even some types of cancer [10,11,12,13]. Each recombinant therapeutic protein has specific physicochemical properties and biological functions, which require customized formulation strategies to preserve structural integrity, improve stability, and minimize potential adverse immunogenic responses in different patients [14]. The complexity of these recombinant proteins lies in their intricate structures, which include post-translational modifications such as glycosylation and phosphorylation, which significantly influence biological and immunogenicity functions [15].

Traditionally, therapeutic formulations include a buffer system to control pH, in addition to stabilizers (e.g., sugars, amino acids), surfactants, and salts [16,17]. However, there is a trend towards unbuffered or self-buffering formulations, in which conventional buffer salts are not added, and the protein itself (or other excipients) is responsible for maintaining the pH of the solution [18]. Here, the term ‘unbuffered/self-buffering’ refers to final drug product formulations viable primarily at high concentrations; buffered systems remain appropriate for low-concentration products and molecules with narrow pH ranges. The advancement of recombinant therapeutic proteins represents a significant contribution to the pharmaceutical sector. Currently, the formulation of these products has gained increasing relevance as a critical factor in ensuring both efficacy and safety in the treatment of a wide range of diseases [19,20]. An understanding of the complexities involved in the formulation of these biological molecules is critical for the developers of new biopharmaceuticals, including so-called biosimilars [21,22]. The latter are highly complex drugs designed to mimic biological drugs whose patent protection has expired but are rigorously regulated with the highest standards to maintain the quality, safety, and efficacy of reference molecules, which, due to their biological characteristics, are not identical [23,24,25,26,27,28].

In recent years, there has been a notable paradigm shift towards the use of buffer-free formulations, aiming to optimize protein stability while minimizing immunogenicity and adverse reactions observed with conventional buffer-containing formulations [29,30,31]. This trend arises from the realization that traditional buffers can not only complicate the manufacturing process, but could also negatively affect the stability of therapeutic proteins during storage and transport [32,33]. However, protein stability is significantly affected by their interaction with excipients, such as polyethylene glycol (PEG) and sugars, but it is essential to maintain protein structure and prolong therapeutic action [34,35]. Research indicates that altering the composition of the buffer can influence protein folding and aggregation, affecting both therapeutic efficacy and the patient’s immune response [36]. A better understanding of these interactions is essential to design formulations that mitigate undesirable immune activation while improving therapeutic efficacy [37]. Therefore, buffer-free formulations are being developed to avoid the detrimental effects of certain buffers and excipients that are known to enhance innate immune responses and activate danger signals in vivo [38].

However, one of the biggest drawbacks with this type of drugs is the high cost of treatments based on these proteins, which can make it unattainable for many low-income patients throughout the world [39,40,41]. In this sense, the possibility that recombinant therapeutic proteins can be replaced by biosimilar drugs is crucial for the accessibility of highly effective and efficient drugs in the treatment of complex diseases for patients with difficulties, either at an economic level or in low-resource countries [42,43]. Although biosimilars contain the same amino acid sequence as their parent products, they can exhibit variations due to differences in manufacturing processes and the use of different excipients that can affect pharmacokinetics and immunogenicity, so rigorous comparability studies are required to ensure the safety and efficacy of the new molecule [26,44]. Critical quality attributes such as structure, biological activity, and clinical outcomes must closely match those of the reference product, highlighting the significant complexities involved in their formulation, production, and characterization [33]. One of the challenges in generating biosimilars lies in the formulation of appropriate expression systems and the optimization of cell culture processes, which can lead to significant variations in post-translational modifications of the proteins produced [45]. To successfully address these challenges, biosimilar developers often turn to the scientific literature and analyzes of expired patents (more than 20 years old) to better understand the formulation approaches employed by their reference products [21,25]. This in-depth analysis of the literature and available patents often serves as a basis for formulating products that maintain the essential characteristics and functionalities of their reference counterparts, while taking advantage of advances in biotechnology to optimize performance [24,46].

However, it should be noted that a critical element is related to the complexities inherent in patented formulations, which often complicates the dissemination of knowledge about formulations in the field of recombinant therapeutic proteins and their respective biosimilars [7,47]. This is because developers often protect certain intricate details of their formulations, which could limit the public availability of information once the patent has expired [48,49]. Different intellectual property regulations may reveal a certain amount of formulation-related data, but complete transparency is rarely achieved, creating a knowledge gap for biosimilar developers looking to replicate successful recombinant therapeutic protein formulations [22,50]. Patents on recombinant therapeutic proteins provide information on the different development steps, but this is usually limited primarily to specific excipients and stabilizers [51]. The exact details of the formulation, including the rationale for choosing specific compounds, are closely guarded, requiring innovation in how developers approach formulation, especially when developing products that must respond to tight deadlines and intense market competition [52,53,54,55].

A fundamental aspect that must be addressed in the production of both recombinant therapeutic proteins and biosimilars derived from these recombinant therapeutic proteins is related to strict regulations, which are essential in the development and approval of new therapeutic proteins [22,27,56]. Regulation of the safety of different compositions of formulations is a multifaceted process that extends beyond traditional toxicological assessments [57]. Agencies such as the US Food and Drug Administration (FDA) and the European Medicines Agency (EMA) emphasize the rigorous evaluation of these formulations to ensure that they are not only safe, but also therapeutically comparable with their reference products in the case of biosimilars and impeccably safe in the case of new molecules entering the pharmaceutical market [14,49]. This involves a comprehensive review of preclinical and clinical data, including how variations in formulation components, as well as different excipients or stabilizers, may affect pharmacokinetic (PK) and pharmacodynamic (PD) profiles [25,58,59]. Regulatory guidelines for biosimilars often require developers to provide comprehensive evidence of similarity, including analytical and clinical data [60]. This scrutiny is crucial, especially considering the sensitive nature of biological therapies, where small changes in formulation can generate significant differences in therapeutic outcomes.

Regulatory frameworks established by leading agencies, such as the FDA and EMA, are critical to assessing the safety of formulation components, including excipients and impurities. These regulatory frameworks establish traditional toxicological assessments and methodologies related to computational modeling and risk assessment approaches and artificial intelligence [22,27,61,62]. To ensure the safety and efficacy of new and biosimilar therapeutic products, regulatory bodies require a detailed understanding of how the components of the formulation interact, including the potential adverse effects of impurities and their implications for biologic immunogenicity [63,64]. Recent guidelines emphasize risk characterization for all components of the formulation, implying the need for systematic assessments incorporating in silico and in vitro models along with clinical data to predict toxicity profiles and ensure patient safety [62,65]. As the formulation landscape evolves for buffer-free recombinant therapeutic proteins, so do regulatory expectations, which encompass rigorous computational analysis to corroborate the safety and purity of the components without the traditional burden of cell culture and animal studies [66]. The safety classification considers the potential for immunogenicity, systemic absorption, and unexpected interactions with endogenous proteins [27]. It has become standard practice to evaluate formulations not only from the perspective of immediate biological impact, but also in terms of chronic exposure and potential long-term adverse events [67].

This article provides, based on a comprehensive documentary analysis of scientific literature, a critical and updated review of formulation technologies applied to recombinant therapeutic proteins. This review focuses on the development, application, and evaluation of buffer-free formulations with a comparative perspective between buffered and non-buffered formulations. Throughout this review, buffer-free/self-buffering’ refers to final drug product designs feasible mainly at high protein concentrations; this approach does not apply to upstream cell culture/expression and is not universally suitable at low concentrations. The Progressive Transition from traditional buffered formulations to Minimal approaches based on protein Self-Buffered Excipients and strategically selected excipients, the regulations governing these formulations (FDA/EMA), and intellectual property rights (patents). It also delves into the importance and safety impact of commonly used excipients in buffered and non-buffered formulations (amino acids, sugars, polyols, surfactants, and antioxidants), classifying them according to their toxicological profile and regulatory approval for parenteral use.

From a regulatory perspective, the requirements imposed by agencies such as the FDA and the EMA are examined, highlighting the growing acceptance of simplified formulations, provided comparability in quality, safety, and immunogenicity is demonstrated, especially in the context of biosimilars. A detailed discussion on the legal and intellectual property implications faced by developers is included, given the limited transparency in the disclosure of formulations protected by expired patents. Finally, an analytical compendium of FDA and EMA-approved formulations for recombinant therapeutic proteins between 2020 and 2025 is presented, highlighting emblematic cases, innovations in excipients, and patterns of transition to buffer-free formulations. These trends are contextualized in relation to emerging technological platforms and current industrial challenges in biomanufacturing, purification, and scale-up.

## 2. Documentary Methodology

Since this research is a review, it is not necessary to describe the methodology as such, but for greater clarification on the selection of the different documents and to proceed with their validation, a framework of the different steps used in the documentary review is provided. It starts from a documentary bibliographic search focused on the identified keywords. The methodology framework is adapted from the document review process (PSALSAR) and specifically refined for recombinant therapeutic protein formulations, with an emphasis on buffer-free strategies, regulatory aspects, and intellectual property [68,69].

The methodology steps are divided into six sections, which are explained below.

○ Protocol Definition: Defines clear research objectives, focusing on buffer-free formulations, regulatory guidelines (FDA, EMA), safety profiles, and intellectual property challenges.
○ Document Search: Systematic search of scientific databases (PubMed, Scopus, Web of Science), patent databases (USPTO, EPO, Derwent), regulatory databases (FDA Drugs@FDA, EMA EPAR) and complementary searches in Google Scholar. Coverage from 2020 to 2025.
○ Critical evaluation: Selection and quality assessment of sources according to predefined inclusion and exclusion criteria, which can be seen in Table 1.

○ Content Synthesis: Extraction and synthesis of relevant information in structured tables and descriptive sections, organized by topics: technological innovation, formulation strategies, excipient safety, regulatory compliance, and intellectual property challenges.
○ Analytical Interpretation: Comparative analysis of buffered and unbuffered formulations, technological platforms, excipient classification, and regulatory trends.
○ Report Generation: Manuscript development, including sections such as introduction, detailed methodology, results, in-depth discussion, and clearly articulated conclusions.

Furthermore, Table 2 shows the details of the methodology with its different phases, the description of the activities, the resources, and databases used to obtain the different documents, and the results and deliverables.

The methodological process began with an exploratory phase based on the research objective, in which representative keywords of the thematic domain addressed in this systematic review were defined. These keywords were selected for their high scientific relevance, their ability to effectively delimit the study universe, and their methodological usefulness in establishing inclusion and exclusion criteria, in accordance with the principles of transparency and reproducibility established by the PRISMA methodology. To guarantee effective and specific document retrieval during the identification phase, a set of strategic keywords was defined that allowed the search to be precisely and systematically guided through scientific databases and search engines with high scientific visibility such as Scopus, Web of Science, Science Direct and Google Scholar, Core Collections, Science Direct, Compendex, Derwent, Google Scholar, Innovation Index, and GeoIndex. These sources were complemented by interdisciplinary research tools that expanded the coverage and precision of the results obtained. To this end, both controlled and uncontrolled terms were integrated using Boolean operators and truncation strategies. The keywords used were: “Recombinant therapeutic proteins”; “buffer-free formulations”; “buffer formulations”; “protein formulation strategies”; “high-concentration subcutaneous biologics”; “regulation frameworks”; “Food and Drug Administration (FDA)”; “European Medicines Agency (EMA)”; “excipient safety classification”; “immunogenicity”; “biosimilars”; “biosimilar formulation”; “pharmaceutical innovation”; “Intellectual property strategies (IP)”. Each of these words was selected for its relevance using “AND” and “OR”, to capture the central dynamics of the study objective, covering regulatory, technological, clinical and legal aspects related to the development and formulation of biosimilars. Likewise, they were formulated in English to maximize coverage in the high-visibility scientific databases used [27].

**Figure 1:**
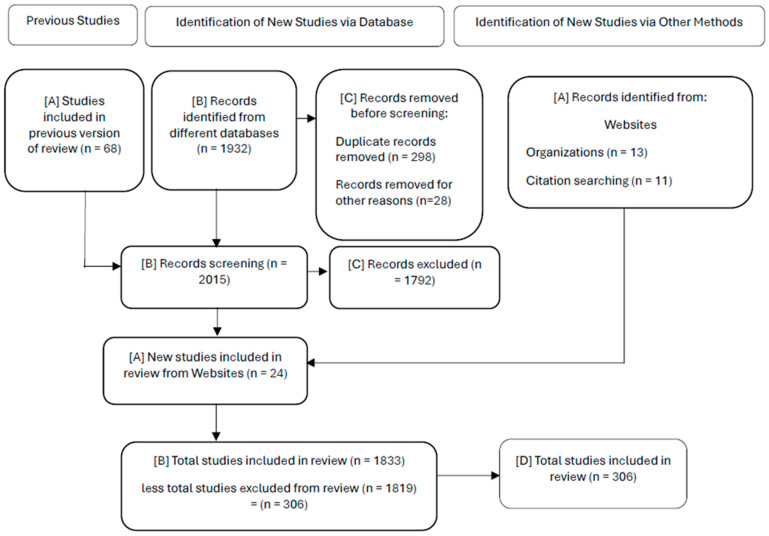
PRISMA-based search process.

The documentary strategy included a systematic review that resulted in a repository of 1932 documents (28 from websites). This repository seeks not only to offer a representative sample of the state of the art on the research topic but also to integrate perspectives from different disciplines with an emphasis on the formulation of biosimilars in the current and future biopharmaceutical context. The review process incorporates a flow chart depicted in Figure 1 illustrating the selection procedure, including the number of studies identified, screened and included, as well as the reasons for exclusion at each stage. This diagram considers previous studies as well as the identification of new studies from databases and the identification of new studies through various search methods. Details can be seen in each box from [A] to [D]. Box [A] represents the studies included in previous versions of the review, as well as the records identified from websites and the new studies included in the review of the website; box [B] represents the records identified from different databases and the cumulative totals, in addition to the review of different records. The total number of studies included in the current review, minus the total number of studies excluded from the review is also shown. The box [C] represents the records eliminated prior to the review, as well as the excluded records. Finally, the box [D] symbolizes the total number of studies included in this article.

Table 3 provides a structured breakdown of how the 306 references included in the review are categorized into four key thematic areas. It reinforces the methodological rigor by quantitatively demonstrating how the evidence was used to inform the thematic synthesis of the review.

Advantages of this “documentary methodology”:

- Systematic Rigor: Structured and reproducible steps ensure methodological transparency and reliability.
- Holistic Integration: Combines technologies, regulatory, safety, and intellectual property perspectives into one comprehensive review.
- Analytical Depth: Allows for in-depth comparative analysis, identifying critical trends and future research needs.
- Flexibility and Applicability: Adaptable across related fields within pharmaceutical development, enabling consistent analytical standards and expanding the potential for future research.

## 3. Recombinant Therapeutic Proteins

Recombinant therapeutic proteins have occupied a prominent place in the biopharmaceutical sector since the approval of recombinant insulin in 1982 [70]. Since this milestone, the repertoire of approved products has grown considerably, now exceeding 100, reflecting notable advances in recombinant DNA technology, protein engineering, and formulation [5,22,71]. These recombinant proteins, which cover a wide range of biological products, such as monoclonal antibodies, hormones, and cytokines, are characterized by having to overcome highly rigorous formulation requirements [55]. These requirements are vital to preserve the structural integrity and biological activity for which they were formulated, but also to minimize immunogenicity, a critical factor that can significantly affect therapeutic efficacy [33].

### 3.1. Mechanisms of Expression of Recombinant Proteins

At the core of the production of recombinant proteins is the genetically modified DNA that encodes the target polypeptide [72]. This DNA must be packaged into an expression vector, usually a plasmid or viral vector, which contains fundamental elements such as a promoter, a ribosomal binding site, and a terminator sequence [73]. The promoter is essential for the initiating of transcription, while the ribosomal binding site is required for efficient translation of RNA into protein [74]. The selection of the promoter and the type of vector can significantly influence the expression levels of the recombinant protein [75]. The process begins with the isolation of a gene of interest, usually by amplification of the polymerase chain reaction or gene synthesis [76]. The gene is then inserted into the expression vector, usually by restriction enzyme digestion or ligation techniques [1]. Once the recombinant DNA construct is formed, it is introduced into the host cell by methods such as transformation or transfection [77]. In bacterial systems, particularly *E. coli*, which is a widely used bacterium for these purposes, heat shock or electroporation are commonly used techniques to facilitate DNA entry [78].

### 3.2. Host Organisms in Protein Production

The choice of host organism plays a fundamental role in the efficiency of recombinant protein expression. In this regard, *E. coli* has established itself as the preferred model organism due to its accelerated growth rate, well-characterized genetics, and established transformation methods [79]. This allows for the rapid and economical production of proteins, especially those that do not require post-translational modifications such as glycosylation [80,81]. The glycosylation patterns present in proteins vary considerably between different cell types, under various physiological conditions, and between biologically similar products, which could affect their efficacy, stability, and potential to elicit a desired immune response [82]. This variability is due to the presence of specific glycosyltransferases, which add different types and structures of glycans to proteins, creating a complex and specific glycosylation profile that can define protein functionality and the potential of glycosylation patterns as biomarkers for the diagnosis and prognosis of disease [83,84]. The ability to transform *E. coli* with plasmid vectors encoding the desired protein ensures the scale of the production process, facilitating large-scale applications in both research and industry [41].

The *E. coli* hosts are often modified to further improve the desired performance and efficiency [81]. Strains such as BL21 (DE3) are specifically designed for high-level expression of recombinant proteins [85,86]. They contain the T7 RNA polymerase system, which enables robust protein production in the presence of T7 promoters [87]. Moreover, *E. coli* serves as an excellent model for studying the folding, solubility, and functional activity of recombinant proteins, providing essential information for downstream applications [88,89]. However, even if *E. coli* remains the most widely used system, alternative hosts have been explored depending on the specific requirements of the protein of interest [90]. Eukaryotic systems, such as yeast (e.g., Saccharomyces cerevisiae), insect cells (baculovirus) and mammalian cell lines, can be used for proteins that require complex post-translational modifications [71,91,92,93]. Yeast systems allow moderate glycosylation and secretion, while mammalian cells provide the most authentic post-translational modification patterns, making them ideal for therapeutic proteins [94].

The efficiency of recombinant protein expression does not depend solely on the structural design of the expression vectors, but is also significantly influenced by the biological characteristics of the selected host system [95,96]. In the case of *E. coli*, it is still widely used due to its rapid growth and high expression yields, but lacks the ability to perform post-translational modifications, such as glycosylation, which are crucial for the functionality of many therapeutic proteins [71,97]. In contrast, mammalian cell lines, particularly CHO cells, provide a suitable cellular environment to produce complex proteins with human-like glycosylation profiles, although at higher production costs and longer development times, which complicates profitability and access [98]. In addition, several strategies are being developed to improve protein expression and therapeutic performance, including incorporation of fusion tags (e.g., SUMO, Fc) to optimize solubility and half-life, as well as the application of genome editing tools such as CRISPR/Cas9 to improve expression stability and product consistency [99,100]. In addition, alternative expression systems such as Pichia (yeast) methylotrophic used in protein production by recombinant DNA techniques are being explored), as well as insect cell lines using baculovirus vectors and genetically modified microalgae as complementary platforms for the production of recombinant proteins, which offer advantages in scalability, cost, and specific post-translational capabilities [71,101].

### 3.3. Advances in Analytical Methodologies for Recombinant Therapeutic Proteins

Analytical methodologies for obtaining recombinant therapeutic proteins are essential to improve their stability and efficacy, while seeking to understand the complex interactions between proteins, excipients, and the patient’s immune system [102,103]. Techniques such as differential scanning calorimetry (DSC) are crucial to analyze protein stability under various buffer conditions, facilitating the optimization of formulations for therapeutic applications [104]. The characterization of protein-excipient interactions can be further enhanced by methods such as native mass spectrometry, which allows for automated and multiplexed screening of ligands in non-volatile buffers [105]. The elimination of conventional buffers minimizes the chances of impurities while reducing the risk of protein aggregation associated with the interaction of ions present in the buffers [106,107]. In addition, the intricate interplay between formulation composition and immunogenicity has been characterized, leading to a variety of more accurate screening techniques and predictive models in silico that directly influence formulation design [108,109,110]. These models have been instrumental in quantifying the risk of innate immune activation by excipients, thus driving the shift towards formulations that rely on minimal excipient content [111,112]. In this sense, certain excipients, such as carboxyvinyl polymers, have been shown to improve antigen delivery and consequently amplify immune activation [113]. This immune activation can alter the innate immune landscape, promote initial threat detection, and potentially lead to enhanced adaptive immune response [38,114]. However, disintegration or modification of components that trigger innate immunity, such as lipid nanoparticles known for their immunogenic properties, highlights a critical approach to optimize therapeutic efficacy without compromising immune tolerance [115]. Since the interaction between innate and adaptive immunity is vital for a balanced response, avoiding harmful buffers tends to produce more favorable immunotherapeutic results [116]. Although the exact mechanism by which specific buffers influence immune responses can vary, minimizing factors that might incite an unwanted immune reaction could contribute to personalized therapeutic strategies that enhance the benefits of treatment [117].

Eliminating buffers in recombinant therapeutic protein formulations arises from the desire to reduce the complexity of the formulation matrix while decreasing interactions that lead to immune activation [19,102]. Some excipients traditionally included in buffer systems can inadvertently act as danger signals, increasing the immunogenicity of therapeutic proteins [55,118]. By adopting a buffer-free approach, manufacturers are now looking to simplify the environment surrounding the active pharmaceutical ingredient to better control issues such as aggregation and degradation [119]. In this context, mechanistic marker-based screening tools are increasingly being used to generate formulations to predict the clinical immunogenicity of these products, ensuring that buffer-free formulations maintain stability and bioactivity [120]. The emerging consensus is that a more agile formulation can produce a superior safety profile without compromising the therapeutic potential of recombinant proteins [37].

### 3.4. Advances in Bioprocessing and Purification

Advances in bioprocessing and purification have highlighted the importance of controlling host cell impurities and other contaminants that can arise during the production of recombinant therapeutic proteins [121,122]. Modern downstream purification methods now incorporate genetic engineering approaches to reduce the presence of host cell proteins, which can act synergistically with excipients to trigger immune responses [123,124]. Strategic gene knockouts have been explored in production cell lines to minimize contamination, which can further improve the viability of buffer-free formulations [12]. Integrating these process improvements into the formulation design has enabled a robust safety classification framework, which ensures that the final products meet regulatory requirements [29]. Therefore, the adoption of advanced purification technologies is closely related to the development of innovative buffer-free formulations that optimize both efficacy and safety [125].

The transition to these simplified formulations marks a significant operational advancement that improves biological manufacturing capacity while aligning with cost-effective production strategies [126]. This shift has been facilitated by various research results aimed at understanding protein stability and the effects of formulation variables on complex protein aggregation and folding processes, focusing on optimizing product formulations based on light and temperature sensitivity [127,128,129,130]. The significance of formulation processes is crucial to ensure not only patient safety in a possible treatment, but also the therapeutic efficacy and overall results of the treatment [130]. An understanding of the complexities involved in the formulation of these biological molecules is critical for the developers of new biopharmaceuticals, including biosimilars [21,28].

### 3.5. Advantages of Recombinant Proteins

The application of recombinant proteins has significantly impacted various fields of life sciences and medicine [5]. One of its main advantages is the ability to produce large amounts of proteins that are often difficult to isolate from natural sources or are present in insufficient quantities [73]. This high yield is crucial for the pharmaceutical industry, as it allows the production of high-demand protein drugs, such as insulin, coagulation factors, and monoclonal antibodies [131]. These proteins can be generated consistently and reproducibly, reducing batch-to-batch variation, which is essential for the intended therapeutic applications [132,133].

Recombinant proteins also facilitate the study of protein function, structure, and interactions, thus contributing to a deeper understanding of biological processes [134]. Recombinant proteins are widely used in drug discovery and validation assays, where their consistent quality plays a crucial role [6,135]. Similarly, the precision of recombinant DNA technology allows the introduction of specific mutations to study structure-activity relationships, generating information that can guide the design of new therapies [136].

It is a fact that the pharmaceutical industry has greatly benefited from the versatility of recombinant proteins in the development of vaccines and therapeutic agents [137,138]. Many modern vaccines, such as the recombinant hepatitis B vaccine, are based on the production of recombinant proteins as the main component that induces an immune response without the risks associated with the use of live pathogens [92,139]. This has allowed advances in vaccine technology with improved safety and efficacy profiles.

### 3.6. Innovative Applications and Platforms in the Pharmaceutical Industry

The most prominent use of recombinant proteins in industry includes monoclonal antibodies, which specifically target antigens associated with diseases, offering specific treatment options for conditions such as cancer, autoimmune disorders, and infectious diseases [140]. The specificity and efficacy of monoclonal antibodies make them invaluable in modern therapeutic regimens.

In addition to antibodies, other recombinant proteins, such as enzymes and hormones, have contributed significantly to therapeutic practices [141]. Recombinant human insulin, developed from genetically modified *E. coli* or yeast, is a cornerstone treatment for diabetes, offering a reliable and safe alternative to animal-derived insulin [6,142]. Likewise, protein-based therapies, such as tissue plasminogen activator (tPA) for thrombolysis, have transformed emergency medicine by facilitating the breakdown of blood clots in patients with acute myocardial infarction [143,144,145,146].

The development and refinement of recombinant protein technologies also drives personalized medicine, an evolving field where treatments are tailored to the individual characteristics of each patient [11,146,147]. The ability to produce proteins tailored to the specific needs of each patient population, leveraging genetic information, offers opportunities for innovative therapeutic strategies that substantially improve outcomes in the treatment of different diseases [79].

The confluence of formulation chemistry, immunology, and protein engineering has led to some “breakthrough platforms”, those designed to generate recombinant therapeutic proteins with PD and PK profiles. Table 4 shows some platforms with their operating mechanisms, advantages, examples, and synergies that occur with buffer-free formulations, which we will see in more detail later [19,148]. All of these platforms provide ionizable groups or protective microenvironments that contribute to formulation self-buffering, especially in the high concentration ranges required for subcutaneous administration. The reduced aggregation and intrinsic structural stability decrease the need for traditional buffered excipients, simplifying the formulation profile and improving injection-site tolerability. These strategies, when integrated with buffer-free approaches, enable the design of safer, more economical, and patient-centered products, aligned with regulatory trends and demands for next-generation biosimilars.

#### Innovative Platforms for Generating Recombinant Therapeutic Proteins with Improved PD and PK

Platforms for generating Recombinant Therapeutic Proteins with improved PD and PK essentially refer to technological systems or strategies that combine advances in formulation chemistry, immunology, and protein engineering to optimize the functioning of therapeutic proteins in the body and their duration [160,161,162]. Basically, protein engineering platforms are used to modify the structure of therapeutic proteins and improve their properties, such as their ability to circulate in the body (PK) and their efficacy at the target site (PD) [163,164]. These modifications may involve improving stability, bioavailability, and activity, in addition to addressing aspects such as immunogenicity and production efficiency [165]. These platforms leverage advances in diverse fields such as protein engineering, formulation chemistry, advanced drug delivery systems, immunology, and synthetic biology, thus optimizing the functionality and longevity of therapeutic proteins in the human body [146].

Some of the main innovative platforms used and grouped by technological category are:

- Protein Engineering Platforms

Protein engineering platforms modify the structure of therapeutic proteins to achieve desired PD and PK properties. For example, recent strategies have focused on the use of deep mutational scanning and CRISPR techniques to design proteins with increased stability and bioactivity, which facilitates improved therapeutic outcomes [166,167]. Novel methods, such as the SAMPLE platform, enable autonomous exploration of protein landscapes, significantly accelerating the protein design process and improving the efficiency of protein engineering with custom features [168]. Furthermore, the application of genetic code expansion technologies allows researchers to introduce non-canonical amino acids, which improves the functional diversity of therapeutic proteins and their delivery capabilities [169]. Table 5 shows different protein engineering platforms and how they modify the protein structure to improve PK/PD.

- Chemical formulation platforms.

The formulation chemistry is critical for the stability and delivery of recombinant proteins [102]. The development of advanced hydrogels and other biocompatible materials not only improves protein solubility and stability, but also prolongs their in vivo release profiles [170]. Furthermore, nanoparticle-based formulations can improve the stability of therapeutic proteins under physiological conditions, thereby improving their bioavailability and therapeutic efficacy [171]. The use of synthetic biology approaches in the formulation of drug delivery vehicles can dramatically improve the efficiency of drug delivery while maintaining their structural integrity [172]. Table 6 shows different formulation chemistry platforms, the mechanisms of optimizing the delivery environment and stability, as well as some examples.

- Advanced Management Systems

Advanced delivery systems facilitate controlled release and targeted delivery of therapeutic proteins to specific tissues, improving their efficacy and reducing side effects. Platforms using genetically modified bacteria have shown promise in drug delivery, as these bacteria can autonomously release therapeutic agents in response to specific environmental triggers [173]. Furthermore, the use of synthetic biology for the design of biosensors allows real-time monitoring and regulation of therapeutic protein release, tailoring treatment to patient needs [174]. The advanced delivery system platforms are shown in Table 7, along with their mechanisms and some examples. These platforms control the release or targeting of proteins.

- Immunology-Based Platforms

Immune modifications are crucial to optimizing the safety and efficacy profile of treatments. Proteins. For example, the use of synthetic receptors and modified immune cells enhances the immune response to cancer therapies, thereby improving treatment outcomes [175,176]. The use of CRISPR/Cas9 technology to enhance antitumor activity by engineered microbes has shown promise in targeting tumor cells and minimizing detrimental immune responses [177]. These immunologically optimized platforms help to personalize therapies to individual patient profiles, thereby improving treatment precision. Table 8 shows immunologically based platforms that modify immune interactions to improve safety and efficacy.

- Synthetic Biology and Next-Generation Engineering Platforms

Synthetic biology integrates diverse engineering disciplines to create multifunctional biological systems capable of producing recombinant proteins with improved properties. Recent advances include the use of modular genetic circuits that allow for more precise control of gene expression, thereby influencing the dynamics of protein production [166,178]. Furthermore, synthetic biology facilitates the development of sophisticated cell-free systems, which provide a more flexible and efficient platform to produce therapeutic proteins without the limitations of cellular contexts [179]. The continued fusion of machine learning with synthetic biology offers potential for predictive modeling and efficient design of therapeutic proteins, accelerating the development process [180,181]. Table 9 shows how state-of-the-art synthetic biology and engineering platforms enable different innovative approaches that combine multiple disciplines.

The technological advances of these innovative platforms are revolutionizing the landscape of the development of recombinant therapeutic proteins, offering safer and more effective treatment options for various diseases. Table 10 provides a summary of each platform, including its mechanisms and impacts on PK/D. The arrows ↑ point up to mean an increase of the impact on PK/PD, while the arrows ↓ point down to mean a decrease.

## 4. Immunogenicity, Excipients, and Differences Between Buffered and Unbuffered Formulations

It is important to distinguish and distinguish between “unbuffered” and simply “citrate-free” or “phosphate-free.” Many modern formulations eliminate phosphate or citrate buffers, but replace them with alternative pH stabilizers such as histidine or acetate. These products are buffered but with a different agent.

### 4.1. Buffered Formulations

In conventional buffered formulations, a buffering agent is added to maintain a balanced pH that optimizes stability and solubility over the shelf life of the product [182,183]. Common buffers for biologics and their pH ranges include phosphate (pH range ~6.5–7.5), citrate (pH ~5–6.5), acetate (pH ~4–5.5), succinate (pH ~5–6), and histidine (pH ~5.5–6.5) [184,185,186,187]. The choice of buffer and pH is critical because proteins are typically less soluble or more prone to aggregation at their isoelectric point [19,188]. Buffers help resist pH changes that might occur due to CO_2_ absorption, interactions with glass or degradation byproducts, and contribute to stability in-use when a product is diluted or meets biological fluids [189,190]. A typical monoclonal antibody formulation might use 10–20 mM histidine or phosphate buffer at pH ~6.0, combined with 50–100 mM NaCl for isotonicity and a non-ionic surfactant to prevent surface adsorption [191,192,193]. Buffered formulations have a long history of success in maintaining protein conformation and preventing aggregation. However, buffers can introduce some drawbacks [188]. Certain buffers (phosphate, citrate) can contribute to instability under stress, were, for example, phosphate can crystallize upon freezing/thawing, causing pH changes, while citrate can increase protein opalescence in some cases [187,194]. The buffer components themselves may be degraded (e.g., citrate may undergo Maillard reactions if sugars are present, or histidine may be oxidized) or interact with container materials [195]. Despite the described drawbacks, buffered formulations remain the standard for most biologics due to their proven ability to control pH-dependent degradation pathways. Most therapeutic proteins approved until 2020 used at least one buffering excipient, then there has been an evolution to unbuffered therapeutic proteins [182]. A high percentage of high concentration mAb products use histidine as a buffering agent, and others use citrate, acetate, or phosphate systems, since buffered formulations are versatile and effective, but increase the excipient load of a drug and can increase immunogenicity [18,196]. A descriptive summary of the different technologies used in conventional buffered formulations is shown in Table 11.

#### 4.1.1. Comparative Stability of Buffered vs. Buffer-Free (Self-Buffering) Formulations

Buffered systems maintain pH within narrow ranges and often minimize pH-driven degradation when the setpoint is close to the protein’s minimum-aggregation pH. However, specific buffers can introduce stress-linked liabilities, for example, phosphate crystallization on freeze, thaw with local pH shifts and citrate-associated opalescence in particular contexts. In contrast, buffer-free/self-buffering drug products reduce ionic-strength-driven interactions and may show equal or lower aggregation and opalescence under agitation and freeze–thaw, especially at high protein concentration. Their limitation is the lack of extrinsic buffering capacity, which places a premium on (i) precise initial pH set during manufacture, (ii) vigilant control of storage and headspace CO_2_, and (iii) compatibility with in-use dilution where relevant [189,190]. Taken together, stability is protein- and context-dependent: buffered formulations are optimal for low-to-moderate concentrations and narrow pH windows, whereas self-buffering can be optimal for high-concentration SC presentations when the protein’s intrinsic titratable groups sustain the pH target.

#### 4.1.2. Immunogenicity in Buffered Formulations

The formulation of recombinant therapeutic proteins has originally been based on established buffer systems (phosphate, histidine, citrate/acetate, etc.) combined with stabilizers (sugars, polyols, surfactants) to maintain pH, prevent aggregation, degradation, and ensure isotonicity [27,37,119,197,198]. However, these stabilizing buffers often add complexity to the formulation process and can present challenges related to compatibility, stability, and final production cost [28,33].

The risk of immunogenicity is primarily due to aggregates/impurities and the effects of excipients, rather than the binary presence/absence of buffers. The implications of immunogenicity in the field of recombinant therapeutic proteins are critical, as small changes in formulation can lead to significant variations in clinical outcomes [67,199]. The development of anti-drug antibodies can negatively affect drug efficacy and safety, complicate the interpretation of clinical data, and require extensive immunogenicity assessments during the development process [200,201,202]. This is especially true for subcutaneously administered therapies, where the local immune response can be enhanced, leading to a higher risk of immunogenicity [203,204,205,206]. Developers should consider the immunogenic potential of their formulations, as observed factors of aggregation and the physicochemical stability of proteins can critically influence the immune response [198,207].

Innovative strategies to reduce the immunogenicity of therapeutic proteins are constantly evolving. Some research highlights different techniques to address the risk of immunogenicity, such as rational design approaches that use nanoparticles to modify the immune response [146,208]. It should be noted that nanoparticles and other delivery platforms are analyzed solely as immunogenicity management strategies and not as substitutes for pH control. However, advances in high-throughput techniques facilitate the evaluation of protein aggregates that could cause undesirable immune responses, thus improving biosimilarity assessment strategies [54]. The presence of impurities that modulate the innate immune response can significantly influence the immunogenic profile of protein therapies, highlighting the importance of formulation analysis and quality control [21,201].

### 4.2. Buffer-Free Formulations

The evolution of recombinant therapeutic protein formulations is toward unbuffered proteins. This refers to the ability to express a protein without additional buffer salts, relying on its own buffering capacity or other harmless excipients to maintain pH [27,209]. Proteins, especially at high concentrations, can resist pH changes because their amino acid residues (such as histidines, lysines, aspartates, etc.) can accept or donate protons and thus act as natural buffers [182,210]. This concept was demonstrated in studies of concentrated monoclonal antibody solutions, where at concentrations above ~20 mg/mL, mAbs already exhibit a buffering capacity [211,212]. Unbuffered mAb solutions have shown negligible pH changes in accelerated stability and shaking tests, comparable to or even better than citrate-buffered solutions [213]. Due to a lower aggregate formation and less opalescence than buffered counterparts, it indicates that buffer removal does not compromise stability and, in fact, mitigates some instability phenomena [27,182]. The absence of buffer salts can eliminate specific degradation pathways, as their absence logically prevents catalysis of reactions or the formation of insoluble complexes. However, in many cases, unbuffered formulations still require pH adjustment during manufacturing (e.g., adding a small amount of acid or base to establish the desired initial pH), but without showing buffer residues in the final product [214]. In practice, designs with fewer buffers have so far been applied mainly to products with very high concentrations, where the protein content is sufficient to maintain pH [215]. In lower concentration products, the protein alone may not provide sufficient buffer, and the pH may fluctuate more easily.

Recent studies indicate that buffer-free formulations can achieve comparable stability and activity in certain recombinant therapeutic proteins [102,216]. The emergence of buffer-free formulations illustrates a growing trend aimed at simplifying these processes while improving the stability and shelf-life of therapeutic proteins, ultimately enhancing patient outcomes and reducing production costs [1]. However, important innovations have led to the development and regulatory approval of high concentration buffer-free or citrate-free formulations [27]. These formulations take advantage of the intrinsic self-buffering capacity of the protein itself or employ minimal and non-traditional excipients to optimize patient comfort and stability and possibly a reduction in manufacturing process costs [217]. This transition is due to the recognition that complex formulations can complicate the production process and affect immunogenicity.

Constant innovation in the formulation of technologies allows the effective elimination of traditional buffers without sacrificing product efficacy or stability [218]. This transition is remarkably significant as it reflects a concerted effort that simultaneously benefits the creation of biosimilars that are easier to use and can be manufactured and transported more efficiently [14,219]. Some research highlights the role of polysorbates and the potential for the use of other amphiphilic stabilizers, finding that they can effectively provide the physical and chemical stability necessary for proteins in solution [54]. Thus, they contribute to maintaining solubility and reducing viscosity without the need for buffers, as observed in studies exploring the optimization of monoclonal antibody formulations [129]. Such additives can offer protection against aggregation, a common problem faced by therapeutic proteins during storage or transport, while also improving the compatibility of formulations with different delivery systems [220].

However, it is important to emphasize that, as buffer-free formulations are adopted, potential drawbacks related to immunogenicity and long-term protein stability must also be considered [102]. The removal of traditional buffers can inadvertently cause alterations in the conformational structure of the protein, which may affect its immunological profile [46]. There is an urgent need for innovation in formulation strategies that effectively minimize immunogenic risk and improve protein stability. Research exploring the use of nanoparticles and other novel delivery systems has opened new avenues to achieve these goals [221,222]. Rationally designed nanoparticle platforms can effectively reduce the immunogenicity of therapeutic proteins, indicating the real potential of these advances to achieve safer biosimilars [146]. Therefore, it is crucial to evaluate these buffer-free formulations through rigorous stability studies and immunogenicity assessments during clinical trials. Continuous monitoring and analysis of the safety and efficacy profiles associated with these formulations will be imperative to reassure regulators and healthcare professionals about their beneficial effects [223].

Some of the advantages and a description of the potential benefits of buffer-free formulations are included in Table 12.

### 4.3. Excipients and Safety Profile of Buffer-Free Formulations

There is a transition towards a better understanding of the mechanisms that drive immunogenicity, with particular attention to the role of excipients and contaminants in therapeutic formulations [37,224]. Even when a formulation lacks a formal buffer, it contains other excipients to ensure its stability and usability. Regulatory agencies typically classify excipients according to their safety/toxicology profiles and prior use in approved drugs. In this regard, specific excipients such as trehalose and sucrose are often included in formulations to combat protein aggregation and denaturation [119]. These excipients are known to mitigate degradation pathways through various physicochemical interactions, which are essential for maintaining protein integrity [48]. However, the availability of these data is often insufficient to fully inform formulators, creating significant barriers to optimal development in the biosimilars industry, for example. Key factors, such as the pH stability range of the protein or the precise concentrations of stabilizers, are often not disclosed, becoming a bottleneck to innovation [50,54].

Incorporation of innovative polymeric excipients into buffer-free formulations is emerging as an important strategy to achieve protein stabilization without traditional buffers [225,226]. These specialized biopolymers provide a microenvironment that can protect proteins from denaturation and aggregation, while minimizing interference with biological activity [227]. Recent studies have shown that the use of such excipients can create a self-stabilizing matrix that preserves the native conformation of the protein under stress conditions [228]. Furthermore, the integration of polymer science into the design of protein formulations is opening new avenues to manage liquid stability and long-term storage challenges [102]. This development represents an important convergence between materials science and biopharmaceutical formulation technology, ultimately contributing to safer and more robust therapeutic products.

Table 13 illustrates some of the most common excipients in buffer-free or low-excipient formulations and their safety classifications.

### 4.4. Traditional Buffered Formulations vs. Innovative Unbuffered Formulations

The classification of recombinant therapeutic proteins requires a detailed understanding of their mechanisms of action and their formulation contexts [103]. The approval process requires extensive safety, immunogenicity, and efficacy testing, which will be addressed in the next chapter. Some studies prioritize protein stability based on formulation properties and interactions with excipients [27]. Buffer components have been shown to influence the propensity of proteins to aggregate [119]. However, high-concentration antibody solutions using innovative excipients have been identified, demonstrating that smart formulation strategies can address the need for traditional buffering agents [229].

On the other hand, Table 14 shows some innovations in both self-buffering and buffer-free formulations.

In established formulations, such as those of monoclonal antibodies, particular attention has been paid to their in vivo behavior and the role of the physicochemical properties that affect PK. The approval of these therapeutic agents often depends on the demonstration that changes in formulation, such as those caused by buffer removal, do not adversely affect crucial pharmacokinetic and pharmacodynamic properties [185,230]. Concerns about the use of polysorbate in some formulations have led to a re-evaluation of excipient selection due to its implications for oxidative stability [231].

Table 15 provides a comparison of the most important characteristics of unbuffered versus traditional buffered formulations, summarizing the advantages, limitations, and practical considerations discussed in this report. Comparison of traditional buffered versus unbuffered formulations for therapeutic proteins. This table describes how each approach addresses key aspects such as pH control, stability, patient tolerability, and typical usage scenarios. Buffered formulations offer robust pH maintenance and are well-established, while unbuffered formulations offer simplicity and potential patient-centric advantages, especially in high-concentration SC products. The choice of approach should be tailored to the properties of the protein and the therapeutic context.

### 4.5. Buffer-Free Formulations in Biosimilars

The shift towards buffer-free formulations also presents an interesting avenue for research into biosimilars [22,27]. These developments reflect an emerging trend within biopharmaceuticals that seek to reduce formulation complexity and improve their stability and shelf life, which could lead to improved patient outcomes and lower production costs [129]. Some studies attribute part of these advances to the search for stable and easy-to-use biosimilars that can be manufactured and transported more easily [33]. In particular, the regulatory environment also responds positively to innovations that simplify buffer-free formulations while maintaining safety and efficacy standards, which can facilitate faster approval times for new products, as is the case with biosimilars [48,50]. The successful development of biosimilars not only helps reduce the high costs associated with biological therapies, but also significantly increases patient access to essential treatments [33,232].

Biosimilars are biological drugs that are very similar to an approved reference biological product, without clinically significant differences in efficacy or safety and whose patent protection has expired [26,27]. Traditionally, biosimilars replicate the formulation of the reference product, including buffers to stabilize protein structure and pH [21]. Buffer-free formulations (sometimes referred to as self-buffering formulations) are a strategy where no external pH buffering agents are added; instead, the intrinsic buffering capacity of the protein (ionizable amino acid residues) maintains the pH of the solution [233]. This approach may reduce the discomfort of the injection site associated with certain buffer excipients (e.g., citrate) and potentially simplify formulations [234]. In fact, citrate buffer in subcutaneous biologics such as adalimumab (Humira^®^) has been found to activate pain receptors, driving reformulation [235].

In recent years, several recombinant therapeutic protein biosimilars have adopted buffer-free (or specifically citrate-free) formulations, especially in fields such as immunology and endocrinology, to improve patient experience and differentiate them from reference products [236]. Thanks to advances in recombinant DNA technology, the production of these proteins has become more sophisticated, leading to a wide variety of biosimilars for therapeutic use [5]. The continued growth of the biosimilars market creates opportunities and challenges in terms of access to effective treatments [24,27,43]. Therefore, recombinant proteins form the fundamental basis of many biosimilars, providing the active ingredients necessary for therapeutic interventions.

### 4.6. Understanding Biosimilars Through Some Examples

Recombinant human erythropoietin (rHuEPO): rHuEPO is commonly used for the treatment of anemia associated with chronic kidney disease. Following the expiration of the rHuEPO patent, multiple biosimilars have emerged, such as epoetin alfa and darbepoetin alfa [237]. The importance of analytical similarity between these products is stressed, highlighting that consistent product quality is critical to ensuring therapeutic efficacy and safety [238]. The ability to characterize these products allows for regulatory approval and safe substitution in clinical practice.

Etanercept and its biosimilars: Another critical case study involves biosimilars of etanercept, a tumor necrosis factor inhibitor widely used for the treatment of autoimmune diseases such as rheumatoid arthritis [239]. The transition from the original formulation of etanercept to biosimilars such as YLB113 demonstrates the challenges and successes surrounding the development of biosimilars. Studies indicate that the use of the biosimilar etanercept can lead to substantial reductions in healthcare costs while maintaining clinical efficacy and safety profiles comparable to the reference product [240]. The comparative efficacy of etanercept biosimilars was particularly evaluated in clinical trials, confirming their potential to improve access to treatment for a larger patient population [241].

Adalimumab: This represents another important biological agent for which several biosimilars have been developed [242]. Research on the biosimilar adalimumab HLX03 revealed that its efficacy and safety profiles are comparable to those of the reference product, which has allowed its successful entry into the market in several regions [81]. Methodological approaches to demonstrate pharmacokinetic similarity, adverse event profile, and therapeutic efficacy are vital components for these biosimilars to gain approval and acceptance in the healthcare setting [243].

#### 4.6.1. Analytical Strategies in the Development of Biosimilars. Cost-Effectiveness

Effective analytical strategies are crucial to establish the similarity of biosimilars to reference biologics. Techniques such as mass spectrometry, chromatography, and peptide mapping are commonly employed to identify and compare physicochemical and functional attributes that determine biological activity [244]. This analytical rigor can also help identify potential immunogenic variants, ensuring that biosimilars are as safe and effective as their brand-name counterparts [22,27,245].

Regarding the cost-effectiveness of biosimilars and their impact on the market, their introduction significantly impacts pharmaceutical expenditure and market dynamics [246]. The biosimilars market is expected to contribute to substantial reductions in healthcare care costs and increase patient access to essential therapies [27,247]. These advances align with global healthcare initiatives that aim to make biological treatments more financially accessible, thus improving overall patient outcomes [248]. In addition to cost savings, biosimilars offer greater accessibility to patients who otherwise would not be able to afford the original biologic drugs [24]. By creating more cost-effective alternatives to established therapies, biosimilars can play a pivotal role in expanding access to treatment, especially for chronic diseases that require long-term treatment [249].

#### 4.6.2. Recombinant Proteins Approved Between 2020 and July 2025

Adalimumab biosimilar is a Buffer-free formulation and the most prominent example in immunology. In the monoclonal antibody (mAb) category, adalimumab was one of the first cases where the buffer (citrate) was eliminated to alleviate injection pain [57]. In the field of endocrinology, long-acting insulin analogs have long been formulated without buffers (insulin glargine at pH 4), illustrating the precedent of unbuffered stability [250,251].

On the other hand, Table 16 shows the most recent approved recombinant therapeutic proteins between 2020 and 11 July 2025, sorted by FDA approval date (newest first). Each entry is cross-checked against the primary sources. FDA Status (BLA #; Approval Date), where BLA # is the FDA’s “Biologic License Application” number assigned to that product when it was approved. The approval Date is the calendar date on which the FDA granted that BLA and the product became licensed in the US.

EMA Status (EPAR #; Authorization Date), where EPAR #: The European Medicines Agency’s “European Public Assessment Report” number for that product. Authorization Date is the date on which the EMA (via a European Commission decision) granted the marketing authorization in the EU. The Authorization

In this context, the common name or international nonproprietary name (INN) of the active ingredient is used, i.e., the official chemical or pharmacological name of the medicine, while the “brand name” (or trade name) is the commercial name under which the company markets it.

## 5. IP Rights in the Protection of Recombinant and Biosimilar Therapeutic Proteins

IP related to the development and commercialization of recombinant therapeutic proteins is a key element in protecting the knowledge generated by the manufacturers of these proteins. However, protection of IP rights, primarily through patents, can also pose significant barriers to collaborative research and, in some cases, hinder the advancement and development of innovations [252,253,254]. Biosimilars are often caught in this confusion, as they must navigate the complexities of entanglement and the different strategies used by the companies that own reference biomolecules seeking to protect patented formulations and methods [27,255]. However, while IP rights are necessary and important due to the investments behind the design and development of each recombinant therapeutic protein, they can also hinder scientific collaboration and knowledge sharing, especially when combined with strict patent laws protecting the interests of the original developers [24,256].

### 5.1. IP Rights—Importance of Patents

Intellectual property rights, particularly patents, play an essential role in protecting innovations in recombinant protein formulations. Patents can cover various aspects of biological products, such as specific genetic sequences, production methods, formulations, and new therapeutic uses [27]. The ramifications of these IP barriers can manifest development delays and ultimately higher costs as developers struggle to balance scientific innovation with the legal parameters dictated by existing patents held by original formulation manufacturers [257]. This protection greatly inhibits the collaborative exchange of vital information between researchers and formulators, which could slow the progress of innovative therapeutic strategies within both recombinant proteins and biosimilars [27]. Developers rely on patents and the publicly available scientific literature for information on successful formulations. However, precise details about unique concentrations, stabilizers, or ratios often remain difficult to obtain. The high-level information available through summary documents and regulatory communications does not provide the granular details critical for informed formulation decisions [129]. This lack of transparency not only complicates development efforts, but also poses challenges in ensuring that biosimilars are adequately validated for safety and efficacy.

The characteristics of recombinant proteins, including their biosimilarity to reference products, are based on several compositional factors, such as excipients and stabilizers [33]. Therefore, a systematic review of relevant patents and scientific literature is essential to better understand patented formulations. Databases and publications often provide contextual information related to the function of stabilizing agents, the mechanisms of action of specific excipients, and the complexities involved in maintaining protein stability [14,219]. However, even when general information is accessible, the lack of granular details on formulation dynamics and the intricacies of excipient interactions remains a major barrier to achieving optimal biosimilar development), highlighting the need for innovation and research in the space of formulation technology [22,32].

Due to these high risks, a systematic examination of existing patents and scientific literature becomes essential to better understand patented formulations and formulation technologies used within the biopharmaceutical industry [14]. Published patent documents often contain vital information on the choices of excipients and stabilizing agents used in various formulations, although fragmented [27,258]. Although patent databases can offer some transparency on specific composition details, such references do not always provide complete information on formulation dynamics, complicating the replication of these knowledge bases for biosimilar development [219]. The scientific literature can shed light on the general mechanisms of action associated with stabilizers, elucidating their role in preserving protein structure and function. However, these documents generally also lack the in-depth details that biosimilar developers need to ensure regulatory equivalence with reference products [49,129].

#### Barriers to Information Exchange

Strict patent protection can inhibit collaborative information sharing between researchers and formulators, limiting access to critical data on formulations and processes [259]. Researchers may be reluctant to share findings that could infringe existing patents, leading to a fragmented landscape where innovations can slow down [260]. This restriction is particularly notable in the biosimilars sector, where science-based dialogue is vital to establishing a more advantageous position in the face of entry barriers established by some pharmaceutical companies.

Biosimilar developers face numerous challenges in navigating the complexities of IP laws that protect patented formulations and methods used in the creation of original biopharmaceuticals [255]. The landscape of IP rights is crucial for the development and commercialization of biosimilars, as these rights often determine limitations on information sharing, innovation pathways, and competitive market positioning [22]. IP rights impact formulation development and can even lead to increased costs, as developers balance the imperative for scientific innovation against the restrictive legal parameters prescribed by existing patents [129]. The complexities inherent in the patenting process, such as the duration of exclusivity and the intricacies of the rights claims, often evolve rapidly, requiring developers to be keenly aware of the potential for litigation or infringement lawsuits from reference product manufacturers [60]. Similarly, many patents cover not only active ingredients, but also specific formulations, manufacturing processes, and excipient combinations, creating a tangle of IP complexities that biosimilar developers must navigate [48].

The delicate interaction of IP rights and the competitiveness of biosimilars requires an ongoing dialogue on international harmonization of intellectual property laws and regulations for these medicines to ensure that regulations encourage innovation rather than stifle it [255,261]. Variations in regulatory expectations and intellectual property protections between jurisdictions can create obstacles for developers seeking to interact between multiple markets [46]. An internationally harmonized IP framework for the formulation, development, and commercialization of biosimilars would not only streamline the development process for these medicines, but would also encourage greater investment in research and development worldwide [223].

## 6. Regulations (FDA–EMA) Involved in the Development and Approval of Recombinant and Biosimilar Therapeutic Proteins

Frameworks are essential due to the need for complete transparency on the safety of new recombinant therapeutic protein formulations and biosimilars that may be approved to enter the pharmaceutical market [22,27]. Establishing clear and strict guidelines for the assessment of immunogenicity risk and the characterization of recombinant therapeutic protein formulations and biosimilars leads to more efficient regulatory processes and patient safety outcomes [1].

The development and approval processes for recombinant proteins and their biosimilars are highly influenced by strict regulatory measures from agencies such as the FDA and EMA, which are essential to ensure the safety and efficacy of products in important markets such as the United States and Europe [56,65,262]. These two regulatory agencies are analyzed for their leadership and demanding approval protocols. As the production of these recombinant proteins expands and biosimilars become more popular, the complexity of regulatory requirements becomes more pronounced [21]. In this direction, the FDA and the EMA establish comprehensive guidelines that govern clinical trials, approval processes, and post-marketing surveillance of recombinant therapeutic proteins [236,263].

These regulatory agencies have promoted strict guidelines to ensure that biosimilars offer an efficacy and safety profile comparable to that of their reference products [264]. However, the nuances surrounding trade secret formulations pose unique challenges in terms of regulatory compliance and competitive advantage [33].

### 6.1. Regulatory Challenges in the Development of Recombinant Therapeutic Protein Formulations

Regarding the safety profiles of the formulations, it is evident that they play a crucial role in determining clinical efficacy and defining regulatory approval pathways [146,265]. The safety assessment of therapeutic proteins requires rigorous preclinical and clinical studies, in which formulations undergo rigorous evaluations of potential immunogenic reactions, stability, and overall efficacy, with the goal of ensuring patient safety [33]. Regulatory bodies such as the FDA and EMA require stringent testing to demonstrate that biosimilars offer safety and efficacy profiles comparable to those of reference recombinant therapeutic proteins [1,59,266]. To mitigate these risks, a multifaceted approach is employed that involves identifying factors that influence immunogenicity, such as intrinsic protein properties, formulation conditions, and patient-specific factors [14,219]. Proteins have been shown to generate unexpected immune responses, in some cases leading to adverse events, emphasizing the need for comprehensive evaluations and immunogenicity assessments throughout the therapeutic product life cycle [32,129]. A systematic evaluation of formulations for characteristics that could promote immunogenic responses includes examining the impact of aggregation, post-translational modifications, and the presence of impurities within the formulation [49,60]. Some mechanisms such as protein aggregates and other formulation-related features are specifically activated through mechanistic markers specifically designed to predict the clinical immunogenicity of biologics [48]. The combination of multiple immunogenic signals may increase the risk of adverse immune responses [201,204]. The importance of these advances is crucial, as they offer new avenues to assess and mitigate immunogenicity risks in the early stages of the development process [50].

### 6.2. Regulatory Overview of Recombinant and Biosimilar Proteins—FDA and EMA

The FDA and EMA are responsible for ensuring the safety, efficacy, and quality of new recombinant therapeutic proteins and biosimilars [265,267]. To facilitate this, detailed protocols for clinical trials are provided, which require comprehensive data on pharmacokinetics, pharmacodynamics, and possible adverse effects detected [268].

The FDA’s Biosimilar User Fee Act (BSUFA) facilitates the biosimilar review process, while the EMA guidelines define criteria for demonstrating biosimilarity to reference products [25,262,269]. BPCIA in the United States has been instrumental in outlining a legal framework aimed at fostering the development of biosimilars. The BPCIA was enacted on March 23, 2010, as part of the Patient Protection and Affordable Care Act in the United States regulatory framework for biologics [33,259,270]. This legislation established an abbreviated approval process for biosimilars, facilitating increased market competition and potential cost reductions for both patients and healthcare systems [27,271,272]. The main objective of the BPCIA was to create a regulatory environment similar to that of the Hatch-Waxman Act, which governs the approval of generic drugs, allowing the entry of biosimilars into the pharmaceutical market while ensuring that the safety, efficacy, and quality standards remain strict [273]. The BPCIA seeks to expand access to biological therapies, which are often characterized by their high costs due to the IP that governs them [270]. In this sense, BPCIA plays an important role in promoting the development of biosimilars. The law seeks to reduce drug prices, improve treatment options, and promote economic competition within the biopharmaceutical industry [22,259,271]. Furthermore, BPCIA introduced a negotiation framework to facilitate the exchange of patent information between the originator of the original (reference) drug and the biosimilar applicant in the event of potential litigation [274,275]. Despite its founding intentions, the BPCIA has faced challenges, such as barriers to adoption caused by uncertainties about regulatory pathways and legal and market dynamics that complicate the entry of biosimilars into the market [276]. However, it is fair to note that the law provides some defined approval pathways that can promote the availability of biosimilars while protecting the interests of the original product developers [1].

### 6.3. Approval Processes for Recombinant Proteins and Biosimilars

Although developers disclose some formulation components during drug approval processes, as is the case with the FDA or EMA, the full details often remain confidential, with strict industry regulations protecting this sensitive information [22,33]. This proprietary nature complicates knowledge transfer and formulation practices, as only high-level knowledge is available in public domains, resulting in potential delays or challenges in the development of biosimilars seeking to emulate reference products [1,27].

The formulation of these therapeutic agents is particularly complex due to the multifaceted nature of the proteins involved, and their stability is influenced by many factors, including environmental conditions, concentration levels, pH, and the presence of excipients [102,277]. Developers are often tasked with selecting excipients that not only stabilize the active pharmaceutical ingredient but also improve solubility and prevent complex aggregation [21]. In some cases, the incorporation of polysorbate 80, a common surfactant, has been explored to mitigate aggregation and maintain protein activity [14]. However, their concentration and physicochemical properties can influence the immunogenic potential of the final formulation. Therefore, formulation strategies must be meticulously optimized to achieve a balance between efficacy and safety.

Despite the importance of formulation knowledge in the development of recombinant therapeutic proteins and biosimilars, there remains a notable lack of transparency when it comes to disclosing detailed formulation components. Although biopharmaceutical developers are required to submit formulation data to regulatory bodies such as the FDA and EMA during the approval process, the sensitive nature of this information often leads to a reluctance to disclose details [27]. Many components of these formulations remain confidential, creating barriers to entry for knowledge transfer within the industry. This limitation can create bottlenecks for biosimilar competitors, who may struggle to effectively replicate the nuanced formulations of their reference biologics [219,223]. The reliance on analytical characterization techniques to establish biosimilarity, including physicochemical and biological assessments, adds another layer of complexity, as the selection of appropriate methods crucially influences the outcomes of such assessments [278].

Recent advances in analytical methodologies have developed a more nuanced understanding of the complex interactions facilitated by excipients and their effect on protein behavior [279]. High-resolution mass spectrometry and advanced chromatographic techniques have allowed detailed explorations of post-translational modifications, aggregation profiles, and other critical quality attributes to ensure biosimilarity of therapeutic proteins [26,280]. Such innovations are essential, particularly when considering post-approval market surveillance that requires continuous evaluation of the quality and immunogenicity of the drug [281]. For biosimilars, the post-marketing surveillance landscape is particularly challenging, as consistent performance requires further validation against the original biologic, which requires robust characterization assays to monitor therapeutic consistency [282,283].

The FDA and EMA have established strict guidelines to ensure that biosimilars demonstrate an efficacy and safety profile comparable to that of their reference products, which promotes confidence in their therapeutic equivalence [59]. However, nuances in formulation trade secrets, which are often protected by intellectual property frameworks, pose unique challenges in terms of compliance and gaining a competitive advantage for biosimilar developers [14,219]. Detailing the characterization required to establish biosimilarity often requires access to proprietary data and formulation strategies used by original manufacturers, which are typically unavailable to those seeking to develop biosimilars [284]. Consequently, developers’ reliance on public domain knowledge is fraught with limitations and uncertainties, hampering their ability to create formulations that faithfully replicate reference products.

### 6.4. Regulation of Excipients

The selection of specific active ingredients allowed in recombinant protein formulations as well as in biosimilars is a meticulous process influenced by different regulations [5,146]. Regulatory agencies require that each component of the formulation be justified in terms of safety and efficacy, which requires extensive characterization and stability studies prior to approval [265].

Regulators classify excipients according to their prior use and safety for buffered and unbuffered recombinant protein formulations [285]. Common excipients in injectable formulations (e.g., phosphate, citrate, histidine buffers; polysorbate surfactants; sucrose, trehalose; amino acids such as glycine or arginine) are well established and are generally recognized as safe at controlled doses [286]. However, if a truly novel excipient or an excipient used via a new route is proposed, complementary safety data are required [27,28]. In the case of the WHO biosimilar guidelines, they indicate that a local tolerance study is justified if the biosimilar uses an excipient with little or no prior experience with the intended route [25,287]. In practice, developers tend to use excipients with a history of approved injectables to simplify regulatory approval. Both the FDA and the EMA maintain “inactive ingredient” databases that list the maximum allowed levels of excipients used in approved injectable products, which guides formulation scientists on what is acceptable [288]. In general, the regulatory framework supports innovations in formulation with and without, provided that the quality of the excipient and patient safety are rigorously demonstrated.

## 7. Discussion and Conclusions

### 7.1. Discussion

The advancement of formulation strategies in the pharmaceutical sector, particularly the transition to buffer-free systems for recombinant therapeutic proteins, has important implications for the addressing of immunogenicity and improving therapeutic efficacy. This transition is especially crucial in the development of biosimilars, where even small variations in formulation can significantly influence patient outcomes and the overall success of therapies. The meticulous design of pharmaceutical formulations is crucial not only to stabilize proteins, but also to minimize aggregation, a phenomenon closely linked to increased immunogenicity [27,289].

Buffer-free formulations have emerged as a preferred alternative, showing profound benefits in stabilizing therapeutic proteins and reducing injection site reactions, as demonstrated with adalimumab biosimilars. By eliminating traditional buffers such as citrate, researchers have observed a marked reduction in discomfort in administration, thereby improving the overall patient experience and promoting adherence to treatment [290]. This evolution in formulation reflects a broader trend toward simplification without compromising safety or efficacy. Thorough characterization and validation of these formulations using modern analytical tools are essential to ensure their stability and safety [24,216,291].

However, these advances come with complex regulatory and intellectual property challenges that must be addressed to facilitate greater acceptance and implementation of recombinant proteins and biosimilars in healthcare. The evolving landscape requires a regulatory framework that balances intellectual property rights with the need for greater transparency and scientific collaboration. This is crucial in the context of biosimilars, where achieving precise replicability of complex therapeutic proteins is inherently difficult due to their complex nature [292,293].

Furthermore, studies have shown that the legal and clinical implications of transitioning to buffer-free formulations require a thorough analysis. The proposed frameworks seek to guide future research and industrial development by offering strategies to optimize formulations and thus minimize immunogenic and regulatory challenges [294,295]. This indicates a potential shift not only in formulation practices, but also in the regulatory landscape that governs them, highlighting the need for agility and adaptability in regulatory agencies to keep up with scientific advances.

Current challenges surrounding biosimilar immunogenicity underscore the importance of continued research and development, not only to improve patient-centered outcomes but also to ensure rigorous safety protocols during the preclinical and clinical phases of development. The need for educational initiatives targeting healthcare stakeholders is critical to dispel myths about biosimilar safety, thereby fostering a more favorable environment for their adoption in clinical practice [293,296].

As biopharmaceutical innovations advance, collaboration between academia, industry, and regulatory bodies will be critical to address the numerous challenges associated with biosimilar development. A comprehensive regulatory approach, considering both innovation and patient safety, will be essential to promote the successful integration of these new formulations into therapeutic regimens. Aligning regulatory strategies with scientific innovation will foster an enabling environment for biosimilar market growth, thereby improving patient access to essential therapies [297,298,299].

The phenomenon of protein aggregation is a major concern in the biopharmaceutical sector, particularly because of its direct implications for the immunogenicity of therapeutic proteins. Aggregation can trigger an immune response by which the body produces antibodies against these proteins, affecting their safety and treatment efficacy, especially in chronic therapies where patient tolerance is crucial [27]. The design and development of antibody therapies require careful consideration of several factors, including formulation strategies that can help mitigate the risk of aggregation. For example, the incorporation of specific excipients and the strict control of storage conditions are strategies that improve protein stability and reduce the propensity to aggregation [289].

Recent advances in formulation strategies have demonstrated that the elimination of traditional buffers in formulations, as observed with adalimumab biosimilars, can significantly improve patient comfort during administration. This formulation improvement has shown potential to reduce discomfort at the injection site and could ultimately lead to improved patient adherence [291]. Several studies indicate a marked reduction in immunogenicity when traditional stabilizing agents are replaced by optimized formulations specifically designed for the therapeutic proteins involved [24].

Research has extensively documented the interplay between aggregation and immunogenicity, particularly in the context of recombinant proteins. Some studies highlight that even small alterations in protein formulations can lead to significant variations in patient response to therapies, reinforcing the need for rigorous formulation optimization processes [216]. For example, the role of post-translational modifications (PTMs) in the induction of aggregation has been demonstrated, where misfolding and inappropriate PTM contribute to an increased risk of aggregation and subsequent immunogenic responses [300].

To address these challenges, the development of advanced formulation methodologies, focusing on optimizing the stability of therapeutic peptides in aqueous solutions, has become increasingly relevant [26]. These methodologies often emphasize the need for a deep understanding of protein chemistry and the physicochemical characteristics that influence protein stability and aggregation under various conditions. Understanding the interactions between proteins and their excipients enables more effective formulation strategies that improve drug stability [301].

Further investigations into the mechanics of protein stabilization have revealed the crucial importance of specific interactions between excipients. According to Pan et al., studying protein-excipient interactions using advanced techniques, such as hydrogen-deuterium exchange mass spectrometry, can reveal how excipients mitigate aggregation in protein formulations [59]. These studies have practical implications, particularly in the design of formulations that are less prone to aggregation and can be stored under less stringent conditions without compromising stability.

Furthermore, it has been recognized that aggregation can reduce therapeutic efficacy and improve immunogenicity, causing many formulations to incorporate surfactants such as polysorbate 20 to stabilize proteins in solution [297]. However, understanding the adsorption behavior of surfactants, as demonstrated by research findings such as those of Ren et al., highlights potential complications, as surfactants can adsorb to filters during the final filtration process, reducing their effectiveness and affecting protein stability [292].

In addition, modeling techniques have been developed to predict aggregation kinetics based on molecular dynamics simulations, offering new strategies to preemptively identify and contrast potential aggregation risks in therapeutic proteins [293]. This predictive modeling approach is becoming an essential complement to empirical testing in formulation development, enabling a more proactive and informed approach to protein stability during manufacturing.

Recent studies underscore a significant shift toward buffer-free formulations in the biopharmaceutical landscape, reflecting a strategic evolution aimed at optimizing therapeutic product compositions while maintaining their integrity and efficacy. This trend is particularly relevant in the context of recombinant therapeutic proteins and biosimilars, where the product formulation can directly influence both the stability and safety profile of these biologics. Simplifying formulation components not only optimizes the manufacturing process, but also promotes improved patient adherence and experience [289].

As industry adopts buffer-free formulations, advanced analytical methods have become crucial to ensure thorough characterization of these products. These methods allow researchers to assess the comparative stability and safety of various formulations, specifically the risk of immunogenicity [25,27]. Elimination of traditional buffers, such as citrate or phosphate, has been shown to decrease localized adverse reactions, thus improving patient comfort during the administration of therapeutic agents such as adalimumab and its biosimilars [291]. This approach represents a holistic view of formulation development, combining scientific rigor with patient-centered considerations.

Emerging technologies, such as high-throughput screening (HTS) and sophisticated biochemical characterization techniques, play an essential role in this modern formulation strategy. These technologies not only streamline the development process, but also improve the ability to produce robust formulations that withstand physical and chemical stress [300]. For example, high-throughput techniques allow the simultaneous analysis of numerous formulation variables, allowing the most stable and effective combinations to be identified efficiently Sarin et al., 2024 [216]. In parallel, advanced biochemical assays are vital to monitor protein aggregation status, ensuring the assessment of potential immunogenicity during the development phase [301].

The integration of these innovative approaches requires the establishment of a rigorous evaluation framework that supports the safety and efficacy claims associated with these new formulations [59]. This framework should encompass a deep understanding of protein chemistry, the impact of various excipients, and the physical conditions that contribute to stability [297]. Advanced computational modeling and machine learning could improve this assessment by predicting stability outcomes based on historical data, helping to prevent the identification of formulations prone to problems such as aggregation or degradation [292].

Recent research exemplifies how buffer-less formulations are gaining ground, especially in the development of therapies that require frequent administration. Patient comfort and adherence to therapeutic regimens are critical in chronic treatment protocols, and buffer-less therapeutic protein formulations can result in less pain and a more tolerable injection experience [291,293]. The broad benefits of these formulations reflect a strong recognition of the relationship between formulation chemistry and clinical outcomes.

In addition, special attention is paid to regulatory frameworks that support innovation in formulation practices. Regulatory bodies increasingly recognize the need to adapt their guidelines to reflect emerging evidence supporting buffer-less formulations. These developments require a collaborative dialogue between pharmaceutical developers and regulatory bodies to streamline the approval process while maintaining public safety standards [294]. Therefore, the voice of the academic world becomes crucial in articulating the findings of recent studies that advocate for more flexible and favorable regulatory policies [27].

Immunogenicity remains a key obstacle to the approval and adoption of biosimilars, compounded by the rigorous regulatory review process in jurisdictions. Several studies highlight that demonstrating equivalence to reference products through analytical and functional similarity is crucial for biosimilars. This requires extensive preclinical and clinical evaluations, especially given the complexity of therapeutic proteins [25,289]. Understanding the nuances of immunogenicity, which can vary depending on formulation and manufacturing processes, is essential for the successful development and acceptance of biosimilars by healthcare professionals and patients [216,291].

For biosimilars, regulatory agencies prioritize rigorous equivalence testing standards, reflecting the complex nature of biologics. WHO guidelines emphasize the importance of comprehensive assessments, requiring biosimilars to demons the world-duality attributes comparable to those of their reference products [27]. This perspective is consistent with that of Jarab et al. [296], who advocate detailed structural and functional analyzes to determine biosimilarity [25]. Regulatory pathways often require diverse data formats to assess both clinical efficacy and safety profiles, which can vary significantly between biosimilars, according to the findings of [289,295].

The regulatory landscape is evolving, but it still faces challenges related to immunogenicity. For example, while progress has been made in establishing parameters for clinical studies, regulatory expectations can be inconsistent. This inconsistency reinforces the need for comprehensive clinical evaluations to detect and quantify immunogenicity [26]. Furthermore, the implementation of analytical methods, such as mass spectrometry and advanced protein characterization techniques, allows a regulatorily compliant understanding of how biochemical properties affect immunogenic responses [301].

The implications of immunogenicity go beyond simple regulatory hurdles; they significantly affect ‘physicians and patients’ perceptions of biosimilars. Several studies demonstrate that concerns about safety and efficacy impact the willingness of healthcare professionals’ to adopt biosimilars in treatment regimens [291,297]. Furthermore, previous experiences with biologic originators often influence perceptions and trust in biosimilars, indicating that ongoing education and dialogue on the robustness of biosimilar development processes are essential to improve acceptance among healthcare professionals and patients, as [292,293].

Furthermore, the influence of health policies and economic factors on the adoption of biosimilars cannot be overlooked. The market dynamics, driven by the potential for savings and greater access to biological products, are often contrasted with concerns about immunogenicity [294,295]. Several studies indicate that when healthcare professionals receive the appropriate information and support on the characteristic safety profiles of well-tested biosimilars, their acceptance progressively improves. This change in perception may contribute to the elimination of some of the barriers associated with the adoption of biosimilars [296,302].

The acceptance and integration of biosimilars into clinical practice faces significant barriers, primarily due to misconceptions about their safety and efficacy among healthcare professionals. Therefore, it is essential to establish a robust educational framework aimed at various stakeholders, particularly healthcare professionals. Such initiatives can alleviate concerns and foster a more favorable environment for the routine use of biosimilars in clinical settings [25].

Educational programs should focus on demystifying biosimilars, emphasizing their clinical equivalence to reference products, and describing the rigorous regulatory processes that these treatments require prior to approval. Evidence suggests that increased awareness and understanding among healthcare professionals can improve conversations between patients and healthcare professionals about biosimilars, ultimately contributing to their acceptance. In addition, specialized training and resources should be provided to prescribers and insurers to highlight the economic benefits of biosimilars, which can significantly reduce healthcare costs while maintaining therapeutic effectiveness [216,291].

The participation of clinicians in educational initiatives is especially crucial, as many healthcare professionals are unfamiliar with biosimilars or hesitant to prescribe them. Abitbol and Chu [303] indicate that this reluctance directly affects patients’ acceptance of biosimilars, as patients’ comfort with these options often reflects their healthcare professionals’ confidence [300]. Therefore, educational initiatives should equip healthcare teams, including nurses and pharmacists, with the knowledge to effectively communicate the benefits and similarities of biosimilars to patients, address common concerns, and build trust.

Incorporating comprehensive educational tools, such as informative workshops, webinars, and accessible educational materials, could significantly improve understanding. Using a biosimilar portfolio that includes clear explanations of manufacturing processes and clinical data can facilitate informed conversations between healthcare professionals and patients [26]. Integrating these educational initiatives will help ensure that biosimilars are viewed not only as alternatives to branded biologics but also as equally effective therapeutic options, thereby fostering greater acceptance.

The economic landscape surrounding biosimilars further underscores the urgency of educational initiatives. Bas [27] notes that educating stakeholders about the cost-effectiveness of biosimilars is critical to promoting their adoption in covered treatment plans [291]. Understanding the potential for significant cost savings may drive policy changes in health systems to incorporate biosimilars more routinely. Sathyan et al. [298] suggest that when cost advantages are communicated along with information on clinical outcomes, healthcare professionals are likely to feel more empowered to prescribe these products with confidence [216].

The sustainability and accessibility of biosimilars on the market are determined by a complex interplay of scientific, regulatory, and intellectual property considerations. These elements are crucial in addressing the challenges posed by chronic diseases that require biologic therapy. The evolving biosimilar landscape must focus on improving patient access while ensuring product safety and quality throughout the approval process [25,27,289].

Recent advances in regulatory frameworks have significantly contributed to streamlining biosimilar approval processes, promoting a more inclusive market. Regulatory bodies are adapting to the growing demand for biological products and the need for efficient pathways to facilitate the introduction of biosimilars, while maintaining rigorous standards [301]. As Prabhash et al. [304] point out, these improved frameworks seek to enable faster entry into the market of biosimilars, which can improve patient access to essential treatments [216]. Similarly, they emphasise the importance of regulatory mechanisms to promote market coverage and ensure that therapeutic options remain affordable [300].

A comprehensive review by Luo et al. [305] underlines that the path to the accessibility of biosimilars to the market involves the harmonization of intellectual property rights, ensuring that patents do not unduly inhibit the development and availability of these vital treatments [26]. This reflects the need to strike a balance between protecting innovation and promoting competition in the biosimilar market. Effective regulation requires strict adherence to quality and safety, as well as understanding of the broader economic implications that govern access to these therapies [291,300].

Some research articulates the need for global cooperation in regulatory standards to improve biosimilar availability, advocating for harmonised practices in jurisdictions to facilitate smoother market entry [27]. The WHO guidelines on biosimilars serve as a fundamental reference for countries looking to develop their regulatory frameworks, highlighting possible mechanisms to improve safety and efficacy, while ensuring patient access [289]. This calls for a continued international collaborative effort to standardize regulations, incentivizing the biosimilar industry to comply with comprehensive global quality standards.

Furthermore, continued stakeholder participation, including healthcare professionals, regulatory agencies, and patients, in the educational discourse on biosimilars is critical. Educating healthcare professionals about the scientific basis for biosimilars, their equivalence in safety and efficacy compared to reference products, and the cost benefits they offer could help overcome the current misconceptions [59,297]. This is crucial to encourage healthcare professionals to adopt biosimilars confidently in clinical practice and to encourage patients to accept these alternative treatments.

### 7.2. Conclusions

This article highlights how technological innovation, regulatory convergence, and rigorous risk management are driving the evolution of recombinant therapeutic protein formulations, with a specific focus on the shift towards buffer-free systems

The rise of buffer-free formulations represents a crucial transformation in pharmaceutical development, driven primarily by the need to improve patient outcomes and optimize manufacturing processes. The interaction between advanced analytical technologies and a rigorous evaluation framework demonstrates a concerted effort to ensure safety and efficacy while fostering innovation in the biopharmaceutical industry. As this trend continues to evolve, the commitment to patient-centered practices will undoubtedly drive further advances in the formulation of therapeutic proteins and biosimilars.

In addition, the ICH guidelines play a crucial role in promoting regulatory convergence across different international frameworks, particularly with respect to the evaluation and approval of biosimilars. These guidelines promote a scientific approach that allows for comparability between various biotech products, which is essential for ensuring patient safety and therapeutic efficacy. Specifically, ICH Q5E outlines standards for comparative studies related to biotechnology-derived products, which is instrumental in managing post-approval changes while maintaining product consistency globally [289]. This regulatory framework helps harmonize the expectations of dossiers submitted to regulatory bodies by ensuring rigorous analytical comparability and uniformity in clinical trial designs.

ICH Q8 emphasizes the principles of Quality by Design (QbD), advocating a flexible approach to pharmaceutical development that improves innovation in formulations [26]. The implementation of QbD helps developers understand the quality attributes that impact product performance, thus significantly contributing to the lifecycle management of biosimilars. Additionally, ICH Q9 focuses on quality risk management, which is vital to make informed regulatory decisions; this structured approach is integral in assessing the risk profiles of biosimilars and ensuring that they meet established standards for safety and efficacy [306]. The combined application of these ICH guidelines by the FDA and EMA has facilitated parallel approval processes for several biosimilars, such as adalimumab (e.g., Amgevita/Hyrimoz) and infliximab (e.g., Inflectra/Resima), highlighting a successful regulatory alignment [66].

With respect to AI and machine learning in biopharmaceutical regulation, the FDA has initiated several programs aimed at harnessing these technologies to enhance the evaluation of biologics. A notable effort is the establishment of the FDA’s Emerging Sciences Working Group, which collaborates with various stakeholders to develop predictive modeling techniques, particularly for key concerns such as protein stability and aggregation [307]. These AI-driven tools facilitate immunogenicity assessments by analyzing sequence data and possible T-cell epitope responses [308]. Such methodologies represent a significant advancement in the efficiency and precision of regulatory reviews, which benefits both novel and established biosimilars alike.

Furthermore, the integration of AI into regulatory processes has shown promise in expediting the evaluation of biosimilar formulations, thereby enhancing access to therapies and potentially reducing healthcare costs [295]. The synergy between traditional regulatory pathways and innovative technological approaches indicates a progressive evolution in the landscape of biopharmaceutical regulations, ensuring that biosimilars maintain a high standard of quality and efficacy while navigating complex global markets [309].

The importance of continuous bioprocessing, sustainable manufacturing practices, and robust educational frameworks should be addressed to improve biosimilar acceptance. These combined efforts position buffer-free recombinant protein formulations as a cornerstone of patient-centered biopharmaceutical innovation.

### 7.3. Tendencies

Emerging trends in the development of recombinant therapeutic proteins are redefining manufacturing and regulatory strategies. Continuous bioprocessing is moving from pilot applications to mainstream adoption, offering shorter production cycles, consistent quality, and reduced costs compared to traditional batch processes. When paired with advanced bioreactor designs, these systems enable real-time process monitoring and tighter control, aligning with industry goals for efficiency and sustainability.

Artificial intelligence (AI)-based models can predict product behavior under various environmental conditions, facilitating the optimization of both formulations and manufacturing processes. This innovation is crucial to managing production efficiency and ensuring that therapeutic proteins consistently meet rigorous quality standards [25,289]. These technologies enable the real-time optimization of production parameters, improving the predictive power related to the stability and efficacy of therapeutic proteins, while accelerating the development-to-commercialization process [26,300,301]. Continuous manufacturing systems, complemented by advanced bioreactor designs, have also been shown to be effective, allowing a more scalable production process that reduces costs while improving the quality of biopharmaceutical products [59,297]. Understanding the implications of the role of AI in the biopharmaceutical industry requires understanding the challenges faced by traditional manufacturing processes. These processes are often long and riddled with inefficiencies, which can hinder timely access to critical therapies. The ability of AI to analyze large data sets and identify patterns enables more informed decision-making, which can optimize operations and lead to higher yields and quality [291]. Recent advances in high-throughput screening techniques, facilitated by AI and ML technologies, illustrate how innovative solutions can optimize purification processes, reducing the overall time to market for essential biopharmaceuticals [216].

A major technological shift is the integration of AI-driven predictive stability models, which forecast protein degradation pathways and optimize formulations without the need for prolonged real-time stability testing. Described AI (XAI) protocols are being developed to improve transparency and regulatory trust, ensuring that AI-generated decisions, such as predicting aggregation risks or refining excipient ratios, are scientifically interpretable [293,294].

Other innovations gaining traction include eco-friendly excipients, patient-centric delivery systems (e.g., autoinjectors with precision dosing algorithms) and high-throughput screening platforms coupled with artificial intelligence to accelerate the optimization of the purification process. On the regulatory side, agencies such as the FDA and EMA are piloting adaptive review processes that incorporate digital analytics into dossier evaluation, enabling earlier identification of risks and more efficient approval timelines.

In manufacturing lifecycle management, AI-enabled model predictive control systems are used to dynamically adjust parameters in response to material or environmental changes, improving yield while ensuring compliance with stringent quality standards. This convergence of advanced manufacturing and data-driven oversight marks a clear departure from traditional approaches, signaling a future in which therapeutic protein production is faster, more sustainable, and more responsive to patient needs.

## Figures and Tables

**Table 1:** Inclusion and Exclusion Criteria.

Criteria	Inclusion	Exclusion
Publication Date	2020–2025	Pre-2020
Document Type	Peer-reviewed articles, patents, regulatory approvals	Abstracts, editorials, non-peer-reviewed
Language	English, Spanish	Other languages
Scope	Buffer-free formulations, recombinant proteins	Non-recombinant proteins, irrelevant topics
Technical Focus	Formulation strategies, safety, regulatory, patents	Unrelated clinical studies, basic research

**Table 2:** Comprehensive Methodology.

Phase	Activity Description	Resources/Databases	Outcome/Deliverable
Protocol Definition	Establish research objectives focusing on buffer-free formulations, regulatory guidelines (FDA, EMA), safety profiles, and IP	Research proposal, previous studies	Defined research questions, scope, and specific objectives
Document Search	Systematic search across scientific, patent, and regulatory databases from 2020 to 2025	PubMed, Scopus, Web of Science, USPTO, EPO, Derwent, FDA, EMA	Comprehensive list of relevant publications and documents
Critical Appraisal	Apply inclusion/exclusion criteria to evaluate the quality and relevance of selected documents	Inclusion/Exclusion Criteria Table, Assessment matrices	high-quality curated documents (~150 documents)
Content Synthesis	Extract and synthesize key information structured by themes (technological innovation, formulation strategies, regulatory, safety)	Selected documents, extraction forms	Structured tables, thematic descriptive summaries
Analytical Interpretation	Perform comparative analysis of buffered vs. buffer-free formulations, technological platforms, excipients, regulatory trends	Comparative analysis tools, thematic summaries	Comparison of analytical tables, identification of key trends
Report Generation	Draft manuscript including introduction, detailed methodology, results, discussion, and conclusions	Analysis results, comparative tables, thematic summaries	Completed manuscript prepared for peer-review submission

**Table 3:** Quantitative Analysis of Thematic References (306 Documents).

Thematic Area	Subcategory	Description	Documents (n)
Technological Innovation	Protein engineering platforms	Fc fusion, PASylation, XTENylation, PEGylation	28
	Analytical/formulation techniques	DSC, Native MS, stability assessments	25
	Bioprocessing/purification	Advanced bioprocessing methods	19
	Innovative delivery strategies	Novel formulation/delivery approaches	13
Regulatory Framework	FDA guidelines/approvals	Regulatory submissions and guidance	22
	EMA regulatory perspectives	EMA regulations and evaluation processes	18
	WHO/ICH harmonization	International Council for Harmonization (ICH) and global harmonization efforts	12
Immunogenicity and Safety	Excipient safety/classification	Safety profiles and classifications	23
	Immunogenicity mitigation	Mechanisms and strategies	21
	Buffer-free tolerability	Safety/tolerability evaluations	20
Intellectual Property Challenges	Patent database analyses	USPTO, EPO, Derwent analysis	45
	Patent expiration issues	Transparency, replication challenges	32
	Biosimilar IP case studies	Disputes and legal examples	28

**Table 4:** Innovative Protein Engineering Platforms.

Platform	Mechanism	Advantages	Examples	Synergy with Buffer-Free Formulations	Reference
Fc-Fusion	Fusion with the IgG Fc domain enables FcRn recycling	Extended half-life, enhanced stability, reduced dosing	Etanercept, Aflibercept	It provides intrinsic ionizable residues (His, Asp, Glu) that act as buffer groups at high concentrations (>50–100 mg/mL), stabilizing the pH without the need for exogenous buffer salts.	[149,150]
Albumin Fusion/Binding	Fusion or binding to albumin extends the half-life via FcRn	Prolonged half-life, reduced renal clearance	Albiglutide, Idelvion	Albumin has ionizable groups capable of buffering pH changes, reducing the dependence on buffers to control solubility and aggregation in concentrated formulations.	[151,152]
XTEN Technology	Fusion with synthetic unstructured polypeptide (XTEN)	Customizable half-life, low immunogenicity	VRS-859, Somavaratan	The high density of polar residues acts as a protein buffer, absorbing or releasing protons to maintain stable pH in the absence of conventional buffers.	[153,154]
PASylation	Fusion with Pro-Ala-Ser (PAS) repeats to increase hydrodynamic radius	Improved half-life, biodegradable and non-immunogenic	PASylated IFN-α, PASylated Factor VIII	PAS bands increase the intrinsic buffering capacity as a result of the presence of carboxyl and amino groups, stabilizing pH, and reducing viscosity without the need for buffering agents.	[155]
PEGylation	Chemical conjugation with polyethylene glycol (PEG)	Increased half-life, reduced immunogenicity	Pegfilgrastim, Certolizumab pegol	The hydrophilic, non-ionic mantle of PEG limits ionic interactions and CO_2_ uptake, reducing pH-dependent degradation pathways and the need for buffers to control the protein’s environment.	[67,156,157]
Glycoengineering	Engineering glycosylation patterns to optimize PK/PD	Enhanced PK/PD, modulated effector function	Darbepoetin alfa, Obinutuzumab	Glycans modify the surface charge distribution, providing local cushioning and creating a protective microenvironment that reduces aggregation and maintains pH without traditional buffers.	[158,159]

**Table 5:** Protein Engineering Platforms.

Platform	Mechanism	Examples
PEGylation	Covalent attachment of polyethylene glycol (PEG) chains to proteins to reduce renal clearance and immunogenicity.	Pegfilgrastim, Peginterferon alfa
Fc Fusion Proteins	Fusion of therapeutic protein with the Fc domain of IgG to utilize the FcRn recycling pathway and prolong the half-life.	Etanercept, Epoetin alfa-Fc (darbepoetin-alfa, Aranesp)
Albumin Fusion Proteins	Fusion with albumin or albumin-binding domains for extended half-life via albumin recycling.	Albiglutide, Tanzeum
Glycoengineering	Modification of glycosylation patterns to enhance serum half-life, reduce immunogenicity, or alter receptor binding.	Obinutuzumab (glycoengineered antibody), Darbepoetin alfa
Amino Acid Mutagenesis	Site-specific mutations to increase stability, reduce degradation, or improve receptor interaction.	Insulin analogs (glargine, degludec), Modified interferons
XTENylation	Genetic fusion to XTEN polypeptides, and unstructured hydrophilic sequences, to increase the hydrodynamic radius and half-life.	VRS-317 (insulin-like growth factor fusion)
PASylation	Fusion to Proline, Alanine, and serine (PAS) sequences to mimic PEG-like effects without PEG-related toxicity.	PASylated growth hormone analogs
Multimerization	Generation of multimeric proteins (eg tandem scFvs, bispecific antibodies) to improve avidity, selectivity, and PK properties.	Blinatumomab (BiTEs)

**Table 6:** Formulation Chemistry Platforms.

Platform	Mechanism	Examples
Buffer-free formulations	Traditional buffers to reduce aggregation and improve stability.	Newer botulinum toxin and monoclonal antibody formulations
Lyophilized or spray-dried formulations	Improve stability and allow alternative routes of administration.	Inhaled insulin (Afrezza), freeze-dried vaccines
Stabilizing excipients (e.g., trehalose, polysorbates, sugars)	Prevent aggregation, maintain conformational stability.	Monoclonal antibody formulations
High-concentration formulations (HCF)	For subcutaneous delivery of large protein doses.	Subcutaneous immunoglobulin therapies
Microenvironment modulating formulations	pH-shifting and osmolarity control for local absorption.	SubQ monoclonal antibodies

**Table 7:** Advanced Delivery Systems Platforms.

Platform	Mechanism	Examples
Encapsulation technologies (microspheres, nanoparticles)	Slow release or targeted delivery of proteins.	Lupron Depot (leuprolide) acetate microspheres)
Hydrogel-based delivery systems	Controlled release over time.	Injectable depot formulations
Lipid nanoparticles (LNPs)	Deliver mRNA coding for therapeutic proteins (indirect protein delivery).	mRNA-based therapeutics (e.g., Moderna’s mRNA-encoded antibodies in development)
Transdermal delivery systems	Protein administration via skin with micro-needles or patches.	Still mostly experimental for proteins

**Table 8:** Immunology-Based Platforms.

Platform	Mechanism	Examples
Tolerization protocols	Co-administration of immune modulators to reduce anti-drug antibodies (ADAs).	Treg-inducing co-therapies
De-immunization by epitope removal	Removal or alteration of T-cell epitopes in protein sequences.	De-immunized coagulation factors
Humanization and fully human antibodies	Reduces immunogenicity by using human sequences.	Adalimumab, Pembrolizumab
Nanobody and VHH platforms	Use of single-domain antibodies with distinct PK/PD profiles.	Caplacizumab (Cablivi), ALX-0171 (anti-RSV nanobody)

**Table 9:** Synthetic Biology and Next-Gen Engineering Platforms.

Platform	Mechanism	Examples
Gene editing-based delivery (CRISPR/AAV)	Direct in vivo production of therapeutic proteins through gene therapy.	Hemophilia gene therapies
Cell-based delivery (CAR-T, engineered T cells)	Live cells are engineered to secrete or display therapeutic proteins.	CAR-T therapies, engineered MSCs
Self-amplifying RNA platforms	Replication of RNA inside cells to express proteins over prolonged periods.	Preclinical vaccine work
Artificial protein scaffolds (DARPins, afilibodies)	Non-antibody protein scaffolds engineered for high-affinity binding and novel PK properties.	Abicipar pegol (anti-VEGF) DARPin)

**Table 10:** Summary for Each Platform and Impact in PK/PD.

Platform	Mechanism	Impact on PK/PD
PEGylation	Covalent PEG attachment	↑ Half-life, ↓ renal clearance, ↓ immunogenicity
Glycoengineering	Modify glycan patterns (branching, sialylation, M6P)	↑ Stability, ↓ clearance (ASGPR), ↑ ADCC/CDC
GlycoPEGylation	Site-selective PEGylation via glycans	Combines PEG benefits with controlled glycosylation
Fc-fusion/Albumin-fusion	Genetic fusion to Fc or albumin	↑ half-life through IgG recycling or albumin pathway
XTEN/PASylation	Fusion to hydrophilic polypeptides (XTEN/PAS)	PEG-like sterics without PEG safety issues
Protein scaffolds (DARPins, etc.)	Engineered non-Ig scaffolds	Tailorable binding, novel PK/PD
Nanoparticle encapsulation	Protein-loaded microspheres, hydrogels, LNPs	Controlled release, targeted delivery
Immunological editing	Epitope removal, humanization, and tolerization strategies	↓ ADA, ↑ efficacy

**Table 11:** Conventional Formulation Technologies.

Category	Description
Buffers and pH Control	Phosphate, histidine, citrate and acetate buffers with molarities of 0.01 to 0.10 M are the most common and provide robust pH control in the range of 4.8 to 8.0.
Stabilizers	Sugars/Polyols (sucrose, trehalose, glycerol) to protect against freeze–thaw and dehydration stress.
Surfactants	Polysorbate 20/80 to prevent interfacial aggregation.
Amino acids	Arginine and glycine were used to improve solubility and inhibit aggregation.
Presentation Formats	Liquid: Immediate use is usually refrigerated. Lyophilized: Reconstituted prior to administration; allows for more extreme pH/excipient options.

**Table 12:** Advantages of Buffer-less Formulations.

Advantage	Description
Simplicity	Fewer excipients in the formulation simplifies development and eliminates potential incompatibilities. This aligns with a minimalist formulation philosophy that uses only what is necessary to stabilize the protein.
Reduced Injection Site Pain	Eliminating acidic solutions such as citrate can significantly reduce local pain after subcutaneous injection. Patients often tolerate injections better when foreign ionic ingredients are minimized.
Improved Stability in Some Cases	Eliminating a buffer can prevent certain instability issues. For example, buffer salts can sometimes promote protein aggregation or opalescence; one study found that citrate-buffered mAb solutions exhibited higher levels of opalescence and aggregation than unbuffered solutions. Buffer-free formulations also avoid pH disturbances caused by crystallization or buffer degradation (e.g., phosphate precipitation during freezing).
Lower Risk of Excipient Interactions	Without multivalent anions or other buffer components, there is a lower risk of interactions with diluents or leachable from the packaging. For intravenous drugs, a self-buffering concentrate can be diluted in saline without the risk of buffer dilution altering the pH or causing precipitation.
Regulatory Flexibility	The use of fewer excipients means fewer variables to control in the manufacturing process and, potentially, fewer regulatory concerns about the purity or origin of the excipients. A buffer-free formulation could simplify regulatory review, as all components (except the protein) are inert stabilizers.

**Table 13:** Excipients and Safety Profile of Buffer-Free Formulations.

Excipient Type	Description/Safety Profile
Amino Acids (e.g., histidine, arginine, glycine, aspartic acid, proline)	Natural amino acids are generally safe for injectable use. Histidine and arginine are widely used in approved biologics (~82% of high potency mAb formulations). Typically, non-toxic at 1–50 mM. Glycine serves as a stabilizer/tonic in immunoglobulin products. Arginine in high concentrations may have osmotic effects orally but is well tolerated in moderate injectable doses. Recognized as safe by FDA/EMA.
Sugars and Polyols (e.g., sucrose, trehalose, sorbitol, mannitol, glycerol)	Serve as stabilizers (especially in lyophilized or liquid forms to prevent aggregation) and tonicity adjusters. The antiviral agent has been well established as safe for parenteral use. Trehalose and sucrose protect against freeze/thaw or heat stress. Sorbitol and mannitol ensure isotonicity (~5% *w*/*v*) and may stabilize by preferential exclusion. Sorbitol in SC mAbs is well tolerated. Mannitol may crystallize when frozen; amorphous stabilizers are preferred in such cases. All are FDA-listed for injectables.
Salts (e.g., NaCl)	NaCl is commonly added for isotonicity, widely considered safe in small doses (e.g., 9 mg per 1 mL SC injection). Some buffer-free products such as Humira (where mannitol is used) are omitted. Acetate or succinate is used as buffers and not in strict buffer-free formulations. The salts used in biologics are chosen from food/pharma-grade sources.
Surfactants (e.g., polysorbate 80, polysorbate 20, poloxamer 188)	Prevent protein adsorption and interface-induced aggregation. PS-80 appears in >90% of antibody products. Safe at ~0.01–0.1%, although rare hypersensitivity reactions have been noted. Can degrade into particles or peroxides. PS-20 is used in interferon-beta, poloxamer 188 in Hemlibra and Enspryng. These surfactants are pharmacopeia-listed and approved for injectables.
Chelators and Antioxidants (e.g., EDTA, methionine)	Chelators like EDTA (used in ppm levels) bind to trace metals, methionine acts as a sacrificial antioxidant. Rare in minimalist buffer-free designs, but used in specific cases like Cyltezo^®^. EDTA is safe in small quantities (0.1 mg/mL) and used in pediatric vaccines. Methionine is a benign amino acid at ~1–5 mM. Considered GRAS and accepted when justified.

**Table 14:** Innovation in Self-Buffering and Buffer-Free Formulations.

Feature	Description
Protein Self-Buffering	Studies demonstrate that intrinsic titratable groups of antibodies can maintain pH within a therapeutic window, even under stress conditions.
Reduced Aggregation and Opalescence	Buffer-free preparations show significantly less aggregation and opalescence compared to their phosphate-buffered counterparts during the shaking and freeze–thaw cycles.
High-concentration Formats	The transition to formulations of ≥100 mg/mL (essential for subcutaneous administration) has been facilitated by eliminating high-ionic-strength buffers, which can promote viscosity and protein–protein interactions.
Patient Comfort	Citrate buffers are known to cause injection site pain; Replacement with self-buffering proteins or minimal excipients has significantly reduced discomfort.

**Table 15:** Comparison of characteristics and selected biosimilars with buffer-free formulations.

Aspect	Traditional Buffered Formulations	Buffer-Free (Self-Buffering) Formulations	Practical Implications
pH control	The external buffer controls pH within its effective range (e.g., phosphate, citrate, histidine).	Initial pH is set during manufacturing; intrinsic ionizable residues provide limited extrinsic buffering.	Buffers are reliable across concentrations, and self-buffering works best at high protein concentrations or for inherently stable proteins.
Stability	Excellent when pH is optimized; some buffers can cause issues (e.g., phosphate crystallization on freeze/thaw; citrate interactions).	Equal or better for high-concentration proteins; often less aggregation/opalescence compared to buffered counterparts.	Choose buffer-free to reduce the aggregation pathways linked to buffer salts; monitor storage tightly to avoid pH drift.
Injection tolerability (SC)	Depending on the type/level of buffer; acidic citrate may sting and increase pain.	Often superior; removal of citrate reduces injection pain and enables smaller volumes at high concentration.	Patient-centric advantage for SC biologics; “citrate-free” positioning supports comfort claims.
Immunogenicity	Driven more by aggregates/impurities than by “buffer vs. no buffer”; good control can reduce risk.	Comparable to buffered; reduced aggregation may further reduce anti-drug antibody risk; no regulator-attributed issues to absence of buffers to date.	Focus analytical control on aggregate minimization in either approach.
Formulation complexity	More components (buffer + stabilizers/surfactants/salts) increase interaction space but leverage mature platform know-how.	Fewer excipients simplify supply chain/QC; may require deeper protein-focused stability work.	Buffer-free can streamline CMC and messaging; buffered can speed development via established platforms.
Typical use cases	IV products at low–moderate concentration; lyophilized products; proteins with narrow pH stability windows.	High-concentration SC biologics (≥100 mg/mL); IV concentrates intended for immediate dilution; excipient-minimizing designs.	Match approach to the route, concentration, and pH tolerance of API.
Representative buffer-free products (regulatory status)		Adalimumab biosimilars: Cyltezo^®^ (adalimumab-adbm), US 2017; interchangeable 2021; high-conc. citrate-free 2024; EU 2017. Hadlima^®^ (adalimumab-bwwd), US 2019 (50 mg/mL), US 2022 (100 mg/mL citrate-free), EU 2017 (Imraldi^®^). Yuflyma^®^ (adalimumab-aaty), EU 2021 (first citrate-free high-conc. in the EU); US 2023. Simlandi™ (adalimumab-ryvk), US 2024; high-conc., citrate-free, interchangeable. Insulins/GH: Semglee^®^ (insulin glargine-yfgn), unbuffered acid (pH ≈ 4); US 2021 (first biosimilar interchangeable insulin); EU 2018. Omnitrope^®^ (somatropin), lyophilized; minimal/no traditional buffer salts in powder (pH set on reconstitution); EU 2006.	The Buffer-free approach has already been approved in major markets and therapy areas; examples support comfort and simplicity narratives for SC use while maintaining regulatory acceptance.

**Table 16:** List of the most recent approved recombinant therapeutic proteins between 2020 and 11 July 2025.

Product (Brand)	International Non-Property Name (INN)	FDA Status (BLA #; Approval Date)	EMA Status (EPAR #; Authorization Date)	Year
Emrelis	Telisotuzumab Vedotin	BLA 761384; 14 May 2025	Not yet centrally authorized (MAA submitted Q2 2025)	2025
Andembry	Garadacimab	BLA 761367; 19 June 2025	EPAR XXXX; authorized February 2025	2025
Enflonsia	Clesrovimab	BLA 761395; 20 June 2025	MAA under evaluation (no EPAR yet)	2025
Encelto	Revakinagene Tarot	BLA 761402; 21 March 2025	EPAR 1054; authorized March 2025	2025
Alhemo	Concizumab	BLA 761315 s000; 20 December 2024	EU/1/24/1881; authorized 13 December 2024	2024
Anktiva	Nogapendekin Alfa Inbakicept-pmln	BLA 761336; 22 April 2024	MAA accepted 27 January 2025 (under review)	2024
Ebglyss	Lebrikizumab	BLA 761306; 13 September 2024	EPAR 0765; authorized 21 November 2023	2024
Hympavzi	Marstacimab	BLA 761283; 11 October 2024 (label)	EPAR 0976; authorized December 2024	2024
Imdelltra	Tarlatamab	BLA 761259; 16 May 2024	EPAR 0893; authorized July 2024	2024
Kisunla	Donanemab	BLA 761227; 2 July 2024	EPAR 0850; authorized April 2024	2024
Winrevair	Sotatercept	BLA 761201; 26 March 2024	EPAR 0802; authorized March 2024	2024
Omvoh	Mirikizumab	BLA 761287; 31 October 2023	EPAR decision; authorized 8 June 2023	2023
Udenyca On-Body	Pegfilgrastim-cbqv	BLA 761161; 22 December 2023	EPAR 0684; authorized December 2023	2023
Darzalex Faspro	Daratumumab + Hyaluronidase-fihj	BLA 761174; 27 January 2023	EPAR 0642; authorized January 2023	2023
Opdualag	Nivolumab + Relatlimab	BLA 761150; 18 March 2023	EPAR 0453; authorized 15 September 2022	2023
Evkeeza	Evinacumab	BLA 761168; 17 February 2023	EPAR 0560; authorized February 2023	2023
Omvoh	Mirikizumab-mrkz	BLA 761279; 26 October 2023	EPAR; authorized 8 June 2023	2023
Izervay	Avacincaptad pegol	NDA 217225; 4 August 2023	Not central EPAR yet (MAA under evaluation)	2023
Udenyca OnBody	Pegfilgrastim-cbqv	BLA 761039 s 015; 26 December 2023	EPAR 0684; authorized 17 June 2021	2023
Opdualag	Nivolumab + Relatlimab	BLA 761150; 18 March 2023	EPAR 0453; authorized 16 September 2022	2023
Vabysmo	Faricimab-svoa	BLA 761235; 28 January 2022	CHMP positive opinion 21 July 2022; EU conditional authorisation 28 September 2022	2022
Beyfortus	Nirsevimab-alip	STN 125730; 17 July 2023	Conditional authorisation EU (CHMP 24 Mar 2022; EC 25 March 2022)	2023
Spevigo	Spesolimab	BLA 761244; 2 September 2022	CHMP positive opinion 12 October 2022; EU conditional authorisation 12 December 2022	2022
Saphnelo	Anifrolumab	BLA 761056; 16 July 2021	EPAR 0742; authorized 27 January 2022	2021
Lupkynis	Voclosporin	BLA 761048; 22 January 2021	EPAR 0739; authorized 24 February 2021	2021
Tezepelumab-ekko	Tezspire	FDA approved 17 December 2021	CHMP positive opinion 21 July 2022; authorized EU 21 September 2022	2021
Efgartigimod alfa-fcab	Vyvgart	BLA 761195; FDA approved 17 December 2021	MAA submitted Q1 2022 (under evaluation)	2021
Ultomiris	Ravulizumab-cwvz	BLA 761205; 21 December 2021	EPAR 0567; authorized 14 January 2022	2021
Teprotumumab-trbw	Tepezza	BLA 761143; FDA approved 21 January 2020	MAA submitted Q2 2024 (under evaluation)	2020
Empaveli	Pegcetacoplan	BLA 761202; 14 May 2021	EPAR 0578; authorized 24 July 2021	2021
Evusheld	Tixagevimab + Cilgavimab	USA (not full BLA)—Authorized for PrEP on 8 December 2021	EPAR: pre-exposure clearance 25 March 2022	2021
Blenrep	Belantamab mafodotin-blmf	Accelerated approval BLA 761052; 5 August 2020	EPAR 0618; authorized 21 July 2020	2020
Vyepti	Eptinezumab-jjmr	BLA 761017; 21 February 2020	EPAR 0620; authorized 1 April 2021	2020

## Data Availability

All Data is in the article.
